# Par-1 Regulates Tissue Growth by Influencing Hippo Phosphorylation Status and Hippo-Salvador Association

**DOI:** 10.1371/journal.pbio.1001620

**Published:** 2013-08-06

**Authors:** Hong-Ling Huang, Shimin Wang, Meng-Xin Yin, Liang Dong, Chao Wang, Wei Wu, Yi Lu, Miao Feng, Chuanyang Dai, Xiaocan Guo, Li Li, Bin Zhao, Zhaocai Zhou, Hongbin Ji, Jin Jiang, Yun Zhao, Xin-Yuan Liu, Lei Zhang

**Affiliations:** 1 State Key Laboratory of Cell Biology, Institute of Biochemistry and Cell Biology, Shanghai Institutes for Biological Sciences, Chinese Academy of Sciences, Shanghai, P.R. China; 2 Shanghai Ocean University, Shanghai, P.R. China; 3 Department of Developmental Biology, University of Texas Southwestern Medical Center, Dallas, Texas, United States of America; 4 Life Sciences Institute, Zhejiang University, Hangzhou, Zhejiang, P.R. China; 5 Institute of Ageing Research, Hangzhou Normal University, Hangzhou, Zhejiang, P.R. China; University of Zurich, Switzerland

## Abstract

The evolutionarily conserved Hippo (Hpo) signaling pathway plays a pivotal role in organ size control by balancing cell proliferation and cell death. Here, we reported the identification of Par-1 as a regulator of the Hpo signaling pathway using a gain-of-function EP screen in *Drosophila melanogaster*. Overexpression of Par-1 elevated Yorkie activity, resulting in increased Hpo target gene expression and tissue overgrowth, while loss of Par-1 diminished Hpo target gene expression and reduced organ size. We demonstrated that *par-1* functioned downstream of *fat* and *expanded* and upstream of *hpo* and *salvador* (*sav*). In addition, we also found that Par-1 physically interacted with Hpo and Sav and regulated the phosphorylation of Hpo at Ser30 to restrict its activity. Par-1 also inhibited the association of Hpo and Sav, resulting in Sav dephosphorylation and destabilization. Furthermore, we provided evidence that Par-1-induced Hpo regulation is conserved in mammalian cells. Taken together, our findings identified Par-1 as a novel component of the Hpo signaling network.

## Abstract 2

Par-1 regulates the Hippo signaling pathway in *Drosophila melanogaster* by modifying the phosphorylation status of Hippo and also by inhibiting the interaction of Hippo and Salvador.

## Author Summary

An organism's organ size is determined by cell number, the size of each cell, and the distance between cells. All of these factors are controlled by the coordination of different cell signaling pathways and other mechanisms. The Hippo signaling pathway controls organ size by restricting the number of cells that make up the organ. Malfunction of this pathway leads to abnormal overgrowth, and is involved in a large number of human diseases and cancers. We identify here a component of the Hippo pathway, Par-1, which controls tissue growth by negatively regulating the Hippo pathway. We show that overexpression or depletion of Par-1 influences tissue growth in fruit flies via Hippo signaling. Then, by genetic and biochemical experiments, we show that Par-1 interacts with Hippo, regulating the Hippo Ser30 phosphorylation status to alter Hippo activity. In addition, we found that Par-1 regulates Hippo signaling via inhibition of the Hippo-Salvador association in a kinase-dependent fashion. We predict that Par-1 is a potential oncogene and that its regulatory role in Hippo signaling could be conserved.

## Introduction

The control of organ size, which requires the delicate coordination of cell growth, cell proliferation, and cell death, is a fascinating biological process. The identification of the Hippo (Hpo) signaling pathway has shed some light on this biological phenomenon. The Hpo pathway has emerged as an evolutionarily conserved pathway that controls organ size during animal development. It regulates tissue growth by balancing cell proliferation and apoptosis and has also been implicated in stem cell maintenance, tissue homeostasis, and repair [1]–[4]. In addition, it has been reported to play a role in cell contact-dependent growth inhibition [5]. Accumulating evidence has suggested that mutations and malfunctions of the components of the Hpo pathway result in a wide range of human cancers and diseases [6].

The Hpo pathway can be divided into three parts: upstream regulatory inputs, core kinase cassette, and downstream transcriptional output [2]. Core to the Hpo pathway is the kinase cascade, which acts sequentially to inhibit the nuclear translocation and activity of the growth-promoting transcriptional coactivator Yorkie (Yki) or Yap/TAZ (mammalian homologue of *Drosophila* Yki) [7]. The core kinase cascade of the Hpo pathway consists of four tumor suppressor proteins, including two kinases, the serine/threonine Ste20-like kinase Hpo or its mammalian homologues MST1/2 [8]–[12], and the nuclear Dbf-2-related (NDR) family kinase Warts (Wts) or its mammalian homologues LATS1/2 [13],[14], and the scaffold proteins of the kinases, Salvador (Sav) [15],[16] and Mob as tumor suppressors (Mats) [17]. Hpo phosphorylates and activates Wts via the formation of a complex with Sav [15],[18],[19]. Wts functions in a complex with Mats to restrict the nuclear translocation of Yki by phosphorylating Yki at multiple sites [7],[17],[19],[20]. In the absence of suppression from the Hpo pathway, Yki associates with transcription factors, primarily Scalloped (Sd) [20]–[22] and other factors, including Homothorax [23], Teashirt, and Mad [24], in the nucleus to promote proliferation and to inhibit apoptosis by inducing the expression of target genes, such as *bantam*, *cyclinE*, and *diap1* [25]. Comparing the clear linear relationship between the core kinase cassette and the downstream transcriptional output, this pathway is regulated by multiple upstream regulatory branches, such as the Merlin-Expanded(Ex)-Kibra complex [26]–[29], Fat (ft) and Dachsous [30]–[33], Crumbs and the Lgl-Scrib-Dlg complex [34],[35], and, the most recently identified, Echinoid and Tao-1 [36]–[38].

Several recent proteome-wide phosphorylation studies, which have uncovered a large number of previously unknown phosphorylation events in Hpo signaling [39],[40], have indicated the involvement of a large number of unknown participants in the Hpo pathway. To identify novel pathway modulators, we performed a gain-of-function EP screen and identified Par-1 as a novel Hpo pathway regulator. Par-1 is a multifunctional serine/threonine kinase containing an N-terminal conserved catalytic domain, a ubiquitin-associated (UBA) domain adjacent to the catalytic domain, and a kinase associated domain 1 (KA1 domain) within the last 40 amino-acids [41]. Par-1 plays a major role in anterior/posterior (A/P) axis formation and germline determinant polarization, and it also regulates diverse cellular processes, including microtubule dynamics and neuronal polarity [42]–[47]. Although Par-1 is involved in multicellular processes, little is known regarding the function of Par-1 in disease and tumor formation. Hyper-phosphorylation of the microtubule-associated protein Tau by microtubule affinity regulating kinase, the homolog of *Drosophila* Par-1 (MARK) [48], which is activated by upstream kinases, such as LKB1 [49] and Tao-1 [50], results in microtubule depolymerization and abnormal aggregation of Tau in Alzheimer disease. Additionally, MARK4 has been reported to be involved in hepatocellular carcinogenesis and gliomagenesis [51],[52].

In this study, we identified Par-1 as a negative regulator of the Hpo kinase complex. We found that overexpression of Par-1 drove tissue overgrowth and upregulated the expression of Hpo pathway-responsive genes. Moreover, knockdown of Par-1 blocked tissue growth and downregulated the expression of Hpo pathway-responsive genes. We demonstrated that *par-1* functioned downstream of *ex* and *ft* but upstream of *hpo* and *sav*. We also provided evidence that Par-1 associated with the Hpo-Sav complex and regulated the phosphorylation of Hpo at Ser30 to regulate Hpo activity. Furthermore, we found that Par-1 promoted the dissociation of Sav from the Hpo-Sav complex, eventually resulting in Sav dephosphorylation and destabilization. Thus, these results identified Par-1 as a novel regulator of the Hpo signaling pathway and supported a model by which Par-1 regulates Hpo phosphorylation and Hpo-Sav association to control organ growth.

## Results

### Par-1 EP Lines Promote Growth via Hpo Signaling

**Figure 1:**
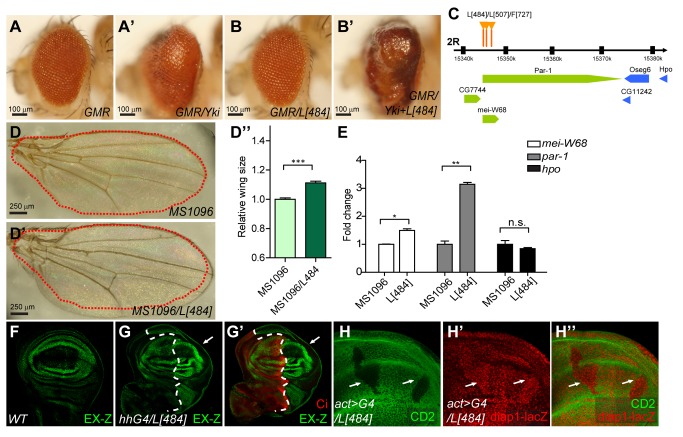
Par-1 P-element insertion lines enlarge organ size and promote Hpo pathway-responsive gene expression. (A–B′) An EP line L[484] enhanced the Yki gain-of-function induced phenotype. Side views of *D. melanogaster* wild-type eyes (A), eyes expressing L[484] (B), eyes expressing Yki (A′), or eyes co-expressing L[484] and Yki (B′), driven by *GMR-Gal4*. (C) Schematic representation of the *par-1* gene locus and P-element insertion sites. The insertion sites of the L[484], L[507], and F[727] EP lines are at the 5′ UTR region of the Par-1 gene (marked by orange arrows). The *par-1* locus is located between *mei-W68* and *hpo*. (D–D″) L[484] promotes adult fly wing growth. Control adult fly wings (D) and wings expressing L[484] (D′) with the wing-specific driver *MS1096*. The red dashed line indicates the size of the control wing. The relative wing size was quantified using an unpaired *t*-test (D″). The results represent the mean ± SEM. ***p<0.001 (*n*>6) for each genotype. (E) Expression of L[484] significantly increased the mRNA levels of Par-1. To detect the level of Par-1 and the transcripts of its neighboring genes, a real-time PCR analysis was performed. All of the results were expressed as the mean ± SEM.*p<0.05, **p<0.01. (F–G′) L[484] promotes *expanded* gene expression. Wild-type *D. melanogaster* third-instar larval wing discs (F) or wing discs expressing L[484] (G–G′) with *hh-Gal4* were immunostained to demonstrate the expression of *Cubitus* (Ci) (Red) and Ex-LacZ (EX-Z) (green). Ci marked the anterior compartment (A-compartment). The arrows indicate the P-compartment. (H–H″) L[484] elevates *diap1* gene expression. Wing discs containing flip-out clones expressing L[484] with *act*>*CD2*>*Gal4* were immunostained to demonstrate the expression of CD2 (red) and *diap1-lacZ* (green). Cells expressing L[484] were indicated by the lack of CD2 expression (indicated by arrows).

To identify novel candidates of the Hpo pathway, we performed an overexpression screen in which flies carrying *GMR-Gal4* and *UAS-Yki* (referred to as *GMR-Yki*) were crossed with a collection of EP lines. Overexpression of *UAS-Yki* posterior to the morphogenetic furrow (MF) under the control of the *GMR-Gal4* driver (*GMR-Yki*) resulted in enlarged eyes (compare Figure 1A′ with 1A), providing a sensitive background for a genetic modifier screen [53]. Each EP line was crossed with *GMR-Yki* flies, and the F1 progeny was screened for an increase in eye size. From more than 10,000 EP lines, we screened numerous lines that enhanced the overgrowth phenotype induced by Yki overexpression. We then analyzed the UAS element insertion sites of these lines and found that the insertion sites of L[484], L[507], and F[727] were all within the 5′ UTR region of the *par-1* gene (Figure 1C). Although these three EP lines did not display an overgrowth phenotype when driven by *GMR-Gal4* in *Drosophila* eyes (compare Figure 1B with 1A, and unpublished data), these lines dramatically enhanced the *GMR-Yki*-induced overgrowth phenotype (compare Figure 1B′ with 1A′, and unpublished data). In addition, the expression of these lines driven under the wing-specific Gal4 driver *MS1096* produced enlarged adult wings (Figure 1D–1D″), indicating that the candidate genes expressed in these lines may play a role in organ size control. To determine whether the UAS element of these lines regulated *par-1* gene expression, real-time PCR analysis was performed for the L[484] line. The mRNA level of *par-1* was significantly upregulated when the L[484] line was crossed with *MS1096*, whereas the mRNA level of genes located proximal to *par-1*, *mei-W68*, and *hpo*, demonstrated a slight or no change (Figure 1E), suggesting that ectopic Par-1 expression could be responsible for the tissue overgrowth phenotype that we observed.

To determine whether overexpression of L[484] promoted tissue growth via Hpo signaling, the L[484] line was expressed under the control of the *hh-Gal4* driver, which drives gene expression in the posterior compartment (P-compartment). As shown in Figure 1G–1G′, *ex-lacZ* (*EX-Z*), an enhancer trap for *ex* [27], was increased in the P-compartment of the wing imaginal disc, suggesting an inhibition of Hpo signaling. Furthermore, the Hpo downstream marker *diap1-lacZ* was also upregulated in the flip-out clones expressing L[484] (Figure 1H–1H″). Briefly, these observations suggested that the expression of the L[484] line promoted tissue growth via Hpo signaling by controlling the expression of Par-1.

### Overexpression of Par-1 Inactivates Hpo Signaling to Induce Tissue Growth in a Kinase-Dependent Manner

**Figure 2:**
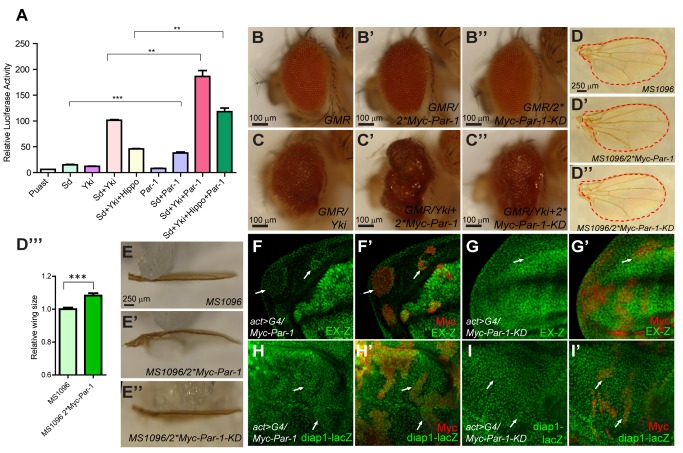
Overexpression of Par-1 triggers tissue overgrowth and inactivates Hpo signaling in a kinase-dependent manner. (A) Par-1 enhances the transcriptional activity of the Yki-Sd complex *in vitro*. S2 cells were transfected with the indicated constructs and the luciferase reporter genes. 48 h after transfection, the cell lysates were harvested and subjected to a dual luciferase assay. Note that Par-1 activates the *3×Sd2-Luc* reporter compared with the control. All of these data were represented as the mean ± SEM. **p<0.01. **p<0.001. (B–C″) Par-1, but not Par-1-KD, synergizes with Yki to trigger tissue overgrowth. Side view of *D. melanogaster* adult eyes: wild-type (B); eyes expressing two copies of *UAS-Par-1* (B′), two copies of *UAS-Par-1-KD* (B″) or *UAS-Yki* (C); or eyes co-expressing *UAS-Yki* and two copies of *UAS-Par-1* (C′) or *UAS-Yki* and two copies of *UAS-Par-1-KD* (C″), driven by GMR-Gal4. (D–E″) Par-1, but not Par-1-KD, induces *Drosophila* wing overgrowth. Dorsal view (D–D″) or side view (E–E″) of the control wings (D, E), wings expressing two copies of *UAS-Par-1* (D′, E′), or wings expressing two copies of *UAS-Par-1-KD* (D″, E″), with *MS1096*. The red dashed line indicated the size of the control wings. The relative wing size was quantified using the unpaired *t*-test (D′″). The results represented the mean ± SEM. *** means *p*<0.001 (*n*>6) for each genotype. Note that the adult wings were bent down in flies that overexpressed Par-1. This phenotype was not observed in the flies that overexpressed Par-1-KD. (F–I′) Par-1, but not Par-1-KD, promotes the Hpo pathway-responsive gene expression. *Drosophila* discs containing flip-out clones expressing *UAS-Myc-Par-1* or *UAS-Myc-Par-1-KD* driven by *act*>*CD2*>*Gal4* were dissected and immunostained with the indicated antibodies. Cells expressing *UAS-Myc-Par-1* or *UAS-Myc-Par-1-KD* transgenes were labeled by Myc tag (indicated by arrows). Note the upregulation of *diap1* and *ex* transcription via ectopic Par-1 expression. Par-1-KD was incapable of inducing *diap1* and *ex* expression.

To verify the functional relationship between Par-1 and the Hpo pathway, a dual luciferase assay, which reflected Sd-Yki transcriptional activity [20], was performed. As shown in Figure 2A, in S2 cells, coexpression of Yki and Sd activated the luciferase reporter gene (*3×Sd2-Luc*), which was greatly promoted by Par-1, indicating that Par-1 enhanced the activity of the Sd-Yki transcriptional complex *in vitro*. To further determine the functional relationship between Par-1 and the Hpo pathway *in vivo*, Myc tagged Par-1 transgenic flies were generated. Consistent with the results in Figure 1A–1B′, overexpression of two copies of *UAS-Myc-Par-1*, using the *GMR-Gal4* driver (referred to as GMR/2*Myc-Par-1), resulted in rough eyes without a discernible overgrowth (compare Figure 2B′ with 2B), while coexpression of *UAS-Myc-Par-1* with *GMR-Yki* enhanced the overgrowth phenotype caused by *GMR-Yki* (compare Figure 2C′ with 2C). Although GMR/2*Myc-Par-1 did not induce a discernible overgrowth phenotype in the eyes (Figure 2B′), the expression of two copies of *UAS-Myc-Par-1*, using the *MS1096* driver (referred to as MS1096/2*Myc-Par-1), resulted in enlarged wings and caused a wing bending-down phenotype, which indicated an expansion of the wing (Figure 2D′″ and compare Figure 2D′ with 2D and 2E′ with 2E). We also found that the relative P-compartment area of the wings expressing *UAS-Par-1*, using the *hh-Gal4* driver, was increased (Figure S1A–S1B). We then examined whether overexpression of Par-1 affected the expression of Hpo pathway-responsive genes. We found that flip-out clones expressing *UAS-Myc-Par-1* in the wing imaginal discs showed upregulated expression of *EX-Z* (Figure 2F–2F′), *diap1-lacZ* (Figure 2H–2H′) and *diap1-GFP3.5* (a *diap1* enhancer element reporter [20], Figure S1C–S1C″), suggesting compromised Hpo signaling activity. Taken together, these results suggested that overexpression of Par-1 promoted tissue overgrowth by inhibiting Hpo pathway activity.

Considering that the Ser/Thr kinase activity of Par-1 was important for its function in polarity regulation, we speculated that the function of Par-1 in Hpo signaling might also be dependent on its kinase activity. To examine this hypothesis, we first constructed a kinase-dead form of Par-1 (Par-1-KD), which contained the T408A and S412A mutations. Par-1, containing these two mutations, was thought to be a kinase inactive mutant because the activation loop of the catalytic domain was disrupted [54]. This was confirmed by an *in vitro* kinase assay in which the kinase activity of Par-1-KD was completely abolished (Figure S1D). Unlike the phenotype observed with the expression of two copies of the Par-1 transgenes, the expression of two copies of the Par-1-KD transgenes had no obvious effect on either eye growth or wing growth and did not dramatically enhance the overgrowth phenotype induced by Yki overexpression (compare Figure 2B″, 2C″ with 2B–2B′, 2C–2C′ and 2D″, 2E″ with 2D–2D′, 2E–2E′). Importantly, to exclude the possibility that the functional variation between Par-1 and Par-1-KD was due to a low expression level of Par-1-KD, we compared the overexpressed Par-1 and Par-1-KD protein levels in both the eye and wing imaginal discs using direct Western blot analysis. We found that the overexpression level of Par-1-KD was higher compared to Par-1, verifying that the functional variation did not result from a low Par-1-KD expression level (Figure S1E–S1F). Furthermore, Par-1-KD failed to elevate Ex and Diap1 expression in flip-out clones (Figure 2G–2G′ and 2I–2I′). Taken together, these observations demonstrated that overexpression of Par-1 promoted tissue overgrowth by promoting the activity of the Sd-Yki complex and upregulating the expression of Hpo pathway-responsive genes in a kinase-dependent fashion.

### Loss of Par-1 Inhibits Tissue Growth by Downregulating Hpo Pathway Targets

**Figure 3:**
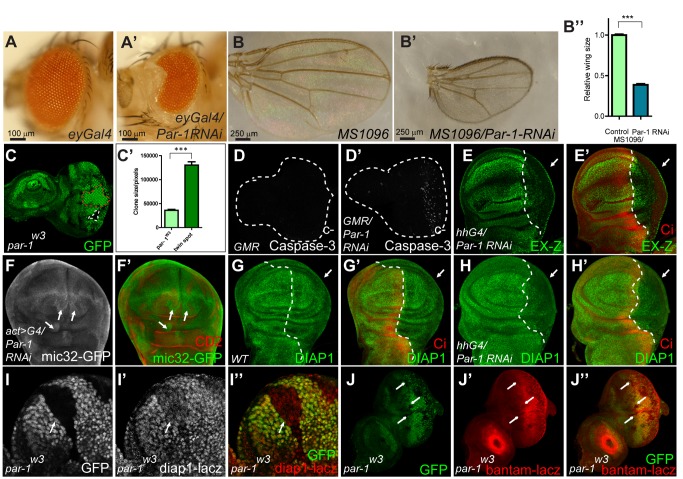
Inactivation of Par-1 reduces organ size and downregulates the Hpo pathway-responsive genes. (A–B″) Inactivation of Par-1 restricts organ growth. Side view of adult fly eyes: wild-type (A) or eyes expressing Par-1 RNAi (A′) under the control of *eyless-Gal4*. Dorsal view of adult wings: wild type (B) or wings expressing Par-1 RNAi (B′) under the control of *MS1096*. The relative wing size was quantified using an unpaired *t*-test (B″). The results represented the mean ± SEM. *** means *p*<0.001 (*n*>6) for each genotype. (C–C′) Knockout of *par-1* restricts cell growth. *Drosophila* third-instar larval eye discs containing *par-1^w3^* clones were dissected. *par-1* mutant clones were marked by the loss of GFP expression, while their twin spots were marked by increased GFP expression. The representative *par-1* mutant clone and its twin spot were separately indicated by the white dashed line and red dashed line (C). The total area of the *par-1* mutant clones or their twin spots within one eye disc were calculated (C′). The results represented the mean ± SEM. *** means *p*<0.001 (*n*>6) for each genotype. (D–D′) Par-1 knockdown initiates apoptosis. *Drosophila* third-instar larval control eye discs (D) or discs expressing Par-1 RNAi (D′) under the control of *GMR-Gal4* were immunostained with a cleaved-caspase-3 antibody. Note that caspase-3 was cleaved to its active form when Par-1 RNAi was expressed. (E–H′) Par-1 knockdown suppresses the expression of the Hpo pathway-responsive genes. Wing discs expressing Par-1 RNAi with *hh-Gal4* (E–E′, H–H′) or with *act*>*CD2*>*Gal4* (F–F′) were immunostained to demonstrate the protein expression levels of *EX-Z* (E–E′), *mic32-GFP* (F–F′), and DIAP1 (G–H′). Cells expressing Par-1 RNAi were labeled either by the lack of Ci or CD2 expression. Note that the expression of EX-Z and DIAP1 was inhibited, whereas the levels of *mic32-GFP* were increased. The arrows indicate the P-compartment or clone regions. (I–J″) Loss of *par-1* disrupts the expression of Hpo pathway-responsive genes. *D. melanogaster* third-instar larval eye discs containing *par-1^W3^* clones were dissected and examined to determine the expression of *diap1-lacZ* (I–I″) and *bantam-lac*Z (J–J″). *par-1* mutant clones were marked by the loss of GFP expression. Note the downregulation of *diap1-lacZ* and *bantam-lacZ* in the absence of Par-1. The clone regions are indicated by arrows.

To determine whether Par-1 is necessary for normal growth, we examined the effect of the loss-of-function of Par-1 on Hpo signaling. By expressing *UAS-Par-1-RNAi* under the control of *eyeless-Gal4 (ey-Gal4)* or *MS1096*, adult eye/wing sizes were reduced (Figure 3A–3B″), suggesting that Par-1 (activity) was required for normal eye and wing development. Par-1 RNAi efficiency was also confirmed by *in vivo* staining, in which Par-1-RNAi transgenes were expressed under the control of *hh-Gal4*. As shown in Figure S2B–S2B′, endogenous Par-1 protein levels were efficiently knocked down by expressing Par-1-RNAi in the P-compartment. In addition, shrinkage of the P-compartment was also observed (Figure S2B–S2B′). To eliminate the concern regarding Par-1 RNAi off-target effects, a second line of Par-1 RNAi (Par-1-RNAi-2), which targets a different region of Par-1, was generated. Par-1-RNAi-2 also efficiently knocked down endogenous Par-1 expression (Figure S2C–S2C′) and restricted wing growth when expressed by *MS1096* (Figure S2D–S2D′). Furthermore, the expression of Par-1-RNAi by *GMR-Gal4* resulted in the detection of caspase-3 in its active (cleaved) form (Figure 3D–3D′), indicating a role for Par-1 in restricting tissue growth by inducing apoptotic cell death.

We further tested whether knockdown of Par-1 resulted in downregulation of Hpo pathway-responsive genes. Expression of either *UAS-Par-1-RNAi* or *UAS-Par-1-RNAi-2* by *hh-Gal4* resulted in diminished levels of *EX-Z*, DIAP1, and *diap1-GFP3.5* and a reduced P-compartment size (Figures 3E–3E′, 3H–3H′, and S2E–S2E′, S2F–S2F″). Consistent with these results, a bantam sensor (*mic32-GFP*) signal was upregulated in Par-1-RNAi flip-out clones (Figure 3F–3F′) or wing discs expressing Par-1-RNAi-2 by *hh-Gal4* (Figure S2G–S2G″), suggesting a restriction of microRNA *bantam* expression by knockdown of Par-1. Thus, this evidence suggested that the inactivation of Par-1 resulted in abnormal growth by antagonizing the expression of Hpo-responsive genes. To further strengthen this conclusion, the expression of Hpo-responsive genes was examined in *par-1^w3^* mosaic clones. As shown in Figure 3I–3I″ and 3J–3J″, in *par-1* null clones, the *diap1* transcriptional level was reduced (Figure 3I–3I″), and *bantam-lacZ* was decreased (Figure 3J–3J″). Importantly, the size of the *par-1* null clones was significantly reduced compared to their twin spots (Figure 3C–3C′), indicating a proliferation disadvantage for *par-1* null clones. Taken together, these observations demonstrated that *par-1* was essential for normal growth and that perturbation of Par-1 expression resulted in growth suppression and apoptosis by stimulating the Hpo pathway.

### *par-1* Acts Downstream of *ex* and *fat* but Upstream of *hpo* in the Hpo Pathway

**Figure 4:**
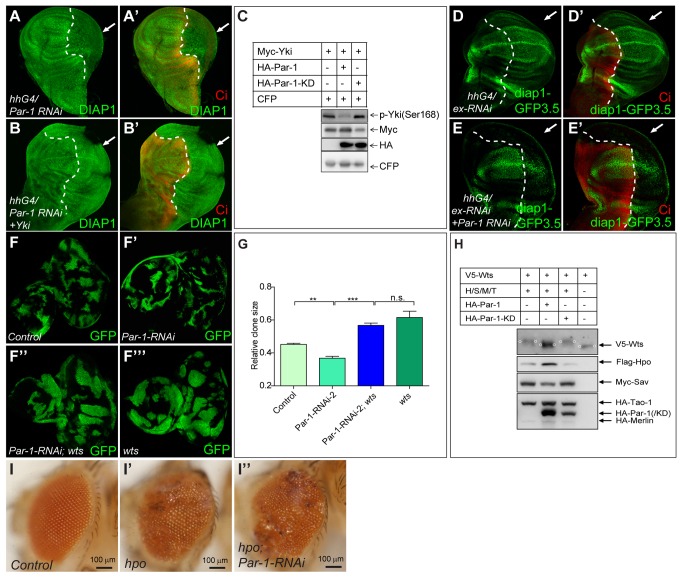
Par-1 functionally interacts with the components of the Hpo pathway. (A–B′) Loss of Par-1 induced a phenotype that was suppressed by Yki overexpression. The protein levels of DIAP1 in the wing discs expressing *UAS-Par-1 RNAi* (A–A′) or coexpressing *UAS-Yki* and *UAS-Par-1 RNAi* (B–B′) with *hh-Gal4* were detected. Note that Yki overexpression overcame the inhibitory effect of Par-1 RNAi on *diap1* expression. The arrows indicate the P-compartment. (C) Par-1 inhibits Yki phosphorylation. S2 cells were transfected with the indicated constructs. Phosphorylated Yki was detected using the p-Yki antibody, which recognizes the phosphorylated site of Yki at Ser168. (D–E′) *ex* functions upstream of *par-1* in the Hpo pathway. Wing discs expressing *UAS-ex RNAi* (D–D′) or coexpressing *UAS-ex RNAi* and *UAS-Par-1 RNAi* (E–E′) with *hh-Gal4* were subjected to immunostaining. The transgene expression regions were marked by the lack of Ci (red) staining and are indicated by arrows. Note that ex RNAi expression resulted in an enlarged P-compartment and increased expression of *diap1-GFP 3.5*, whereas coexpression with Par-1 RNAi fully suppressed these phenotypes. (F–F′″) *wts* functions downstream of *par-1* in the Hpo pathway. Clones were generated using the MARCM system. The genotypes were the following: *ey-flp, Ubi-Gal4, UAS-GFP; FRT82B/FRT82B Gal80* (F), *ey-flp, Ubi-Gal4, UAS-GFP; Par-1-RNAi; FRT82B/FRT82B Gal80* (F′), e*y-flp, Ubi-Gal4, UAS-GFP; Par-1-RNAi; FRT82B wts^latsX1^*/*FRT82B Gal80* (F″), and *ey-flp, Ubi-Gal4, UAS-GFP; FRT82B wts^latsX1^*/*FRT82B Gal80* (F′″). Note that Par-1 RNAi reduced the clone size, whereas *wts^latsX1^* rescued this phenotype. (G) Quantification of the relative clone size. The relative clone size was calculated as the GFP area divided by the entire disc area. All of these data were expressed as the mean ± SEM. **p<0.01. **p<0.001. *n*>5, for each group. (H) Par-1 modulates Wts phosphorylation status. S2 cells were transfected with the indicated plasmids. The cell lysates were harvested and followed by Western blot analysis. Note that the phosphorylation shift of Wts mediated by Hpo/Sav/Merlin/Tao-1 was partially blocked by Par-1 expression. The shifted Wts bands are indicated by the small circles. (I–I″) *par-1* functions upstream of *hpo* in the Hpo pathway. Clones were generated using the MARCM system. The genotypes were the following: *eyflp, ubi-Gal4, UAS-GFP; FRT42D/FRT42D Gal80* (I), *eyflp, ubi-Gal4, UAS-GFP; FRT42D hpo^BF33^/FRT42DGal80* (I′), e*yflp, ubi-Gal4, UAS-GFP; FRT42D hpo^BF33^/FRT42DGal80; Par-1-RNAi* (I″). Note that the Hpo null clones caused tumorous growth, whereas Par-1 RNAi could not rescue this phenotype.

**Figure 5:**
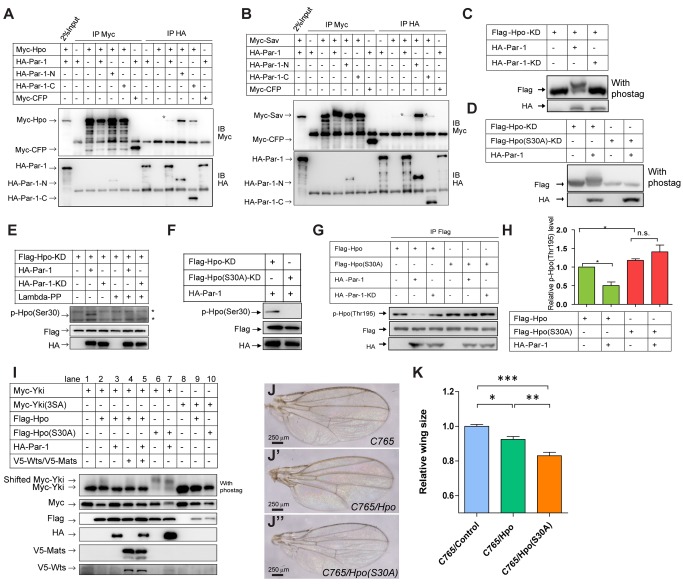
Par-1 interacts with Hpo-Sav and regulates the phosphorylation of Hpo at Ser30. (A–B) Par-1 interacts with Hpo and Sav *in vitro*. S2 cells were transfected with HA-tagged full-length or truncated Par-1 and Hpo (A) or Sav (B) constructs. The cell lysates were immunoprecipitated, followed by Western blot analysis with the indicated antibodies. Note that weak binding (asterisk indicated) between full-length Par-1/Par-1-C and Hpo and Sav were detected, whereas the N-terminal truncation of Par-1, which contained the kinase domain, showed a much stronger interaction signal. (C) Par-1 induces phosphorylation shift of Hpo-KD *in vitro*. S2 cells were transfected with the indicated constructs. The cell lysates were subjected to phosphorylation mobility shift assays. Note the phosphorylation shift of Hpo-KD in the presence of Par-1. Phos-tag was used to enhance the phosphorylation shift (see Materials and Methods for further details). (D) Par-1 regulates phosphorylation of Hpo-KD at Ser30 in S2 cells. S2 cells were transfected with the indicated constructs. The cell lysates were subjected to a phosphorylation mobility shift assay. The Hpo Ser30 site was mutated to an alanine. Note that the Hpo(S30A) mutant did not shift in the presence of Par-1. (E–F) Par-1 induces the phosphorylation of Hpo-KD at Ser30 in S2 cells. S2 cells were transfected with the indicated constructs. The cell lysates were subjected to Western blot analyses. Note that the phospho Hpo(Ser30) antibody could only detect Par-1-induced phosphorylation in the Hpo-KD samples but not in the Hpo(Ser30) mutant samples. The asterisks indicate non-specific bands. Lambda-PP indicates λ-phosphatase. (G) Par-1 inhibits Hpo(Thr195) phosphorylation. S2 cells were transfected with the indicated constructs. The cell lysates were immunoprecipitated, followed by Western blot analyses to detect p-Hpo(Thr195) levels. Note that Par-1 inhibited Hpo(Thr195) phosphorylation in a kinase-dependent manner, whereas the Hpo(S30A) mutant could not be inhibited. (H) Quantification of p-Hpo(Thr195) levels. p-Hpo(Thr195) levels were quantified using densitometry. The results were expressed as the mean ± SEM from three independent experiments. *p<0.05. (I) Hpo(S30A) results in a higher phosphorylation shift of Yki. S2 cells were transfected with the indicated constructs. The cell lysates were subjected to a phosphorylation mobility shift assay. Note that the phosphorylation shift of Yki was enhanced in the presence of Hpo(S30A) and that the Hpo(S30A) mutant was resistant to Par-1 induced Yki dephosphorylation. (J–K) Hpo(S30A) shows enhanced activity compared with wild-type Hpo *in vivo*. Control wings (J) or wings expressing *UAS-Hpo* (J′) or *UAS-Hpo(S30A)* (J″) with *C765* were shown. The relative wing size was quantified using an unpaired *t*-test (K). The results represented the mean ± SEM.*p<0.05, **p<0.01, ***p<0.001 (*n*>6) for each genotype. Note that the Hpo(S30A) flies exhibited smaller wings than the Hpo flies.

Given the findings presented in the previous section, we next determined the functional relationship between Par-1 and Hpo pathway components by identifying the genetic interactions of Par-1 in the Hpo pathway. We first examined whether the function of Par-1 was dependent on the activity of the Sd-Yki transcriptional complex because this complex was the main downstream effector of the Hpo pathway. Although expression of two copies of *UAS-Myc-Par-1* in flip-out clones increased *diap1-lacZ* (Figure S3A–S3A′), no increase in *diap1-lacZ* was detected when *UAS-Yki-RNAi* was coexpressed (Figure S3B–S3B′). Coexpression of *UAS-Sd-RNAi* suppressed the overgrowth phenotype induced by Par-1 overexpression in *Drosophila* wings (Figure S3C–S3C′). In addition, the elevated levels of *diap1* transcription caused by Par-1 overexpression were reverted by coexpression of Sd RNAi (Figure S3D–S3F′). Furthermore, ectopic Yki expression reverted downregulated DIAP1 levels and the shrunken P-compartment phenotype induced by the expression of Par-1 RNAi (Figure 4A–4B′). These results indicated that *par-1* functioned upstream of the Sd-Yki transcription complex in the Hpo pathway. To strengthen this conclusion, the levels of phosphorylated Yki, which reflected Hpo/Wts activity, were examined. As expected, Par-1, but not Par-1-KD, reduced phosphorylated Yki levels (Figure 4C). In addition, Par-1 also inhibited the Hpo/Wts signaling-induced Yki mobility shift (Figure 5I, lanes 2–5). These findings suggested that Par-1 functioned upstream of *yki* to affect the activity of the Sd-Yki transcription complex. We next examined the genetic epistasis between Par-1 and the upstream components of the Hpo pathway. We found that elevated DIAP1 levels and the enlarged P-compartment size (Figure 4D–4D′), resulting from ex RNAi expression by *hh-Gal4*, were suppressed by coexpression of the Par-1 RNAi transgene (Figure 4E–4E′), suggesting that Par-1 functioned downstream of *ex*. In addition, coexpression of Par-1 RNAi suppressed ex RNAi-induced wing overgrowth (Figure S3G–S3G′″). Furthermore, Par-1 RNAi also suppressed Ft RNAi-induced wing overgrowth (Figure S3H–S3H′″). These observations supported the notion that *par-1* functioned downstream of or in parallel to *ex* and *ft*.

We then determined whether Par-1 regulated the Hpo pathway via Wts, which phosphorylates Yki at Ser168 to retain Yki in the cytoplasm [19]. By generating Par-1 RNAi clones in eye discs using the mosaic analysis with a repressible cell marker (MARCM) technique, we found that the size of Par-1 RNAi clones was extremely small compared to that of the control clones (compare Figure 4F′ with 4F), indicating adverse development of the Par-1 RNAi clones. Strikingly, we found that knockout of *wts* rescued the adverse developmental phenotypes of the Par-1 RNAi clones and that the size of the *wts* mutant clones expressing Par-1 RNAi was more comparable to that of the *wts* clones, rather than that of Par-1 RNAi clones (Figure 4G and compare Figure 4F″ with 4F′″). These findings indicated that *par-1* functioned upstream of *wts*. Because Wts was activated and phosphorylated by upstream components of the Hpo pathway, we then tested whether Par-1 regulated Wts phosphorylation. Par-1, but not Par-1-KD, reduced the mobility shift of Wts phosphorylation in the presence of Hpo/Sav/Merlin/Tao-1 (Figure 4H), suggesting that *par-1* functioned upstream of *wts* and regulated Wts phosphorylation in a kinase-dependent manner. We then determined whether Par-1-regulated Hpo signaling was dependent on the activity of Hpo, which restricted tissue growth by phosphorylating Wts. By generating *hpo* mutant clones in *Drosophila* compound eyes using the MARCM system, we found that the ablation of Hpo resulted in tumor outgrowth (compare Figure 4I′ with Figure 4I). Furthermore, we found that Par-1 RNAi was incapable of reverting the growth advantage of *hpo* null clones (compare Figure 4I″ with Figure 4I′), indicating that Par-1 functioned upstream of *hpo* to regulate Hpo signaling.

### Par-1 Interacts with Hpo-Sav and Regulates the Phosphorylation of Hpo at Ser30

**Figure 6:**
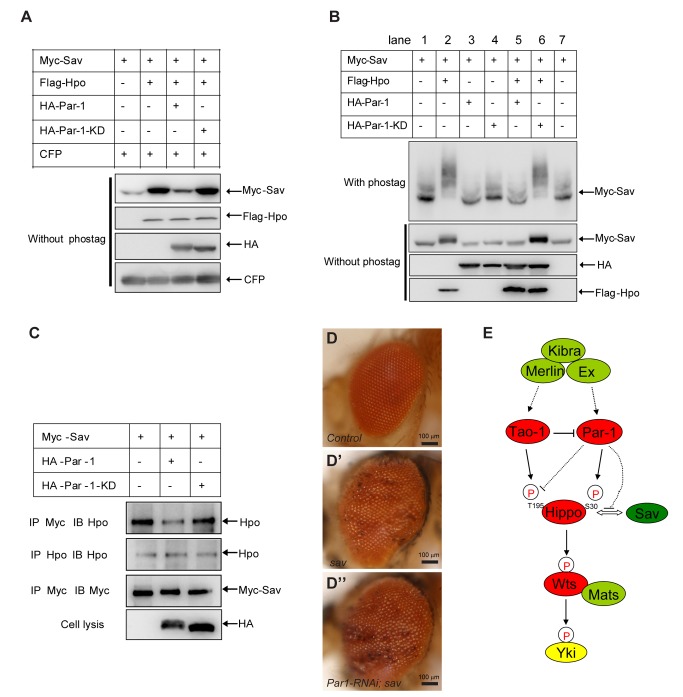
Par-1 disrupts the association of the Hpo-Sav complex in a kinase-dependent manner. (A) Par-1 could destabilize Hpo-induced accumulation of Sav in a kinase-dependent manner. S2 cells were transfected with the indicated constructs followed by a Western blot analysis. CFP served as a loading control. (B) Par-1 inhibits the phosphorylation of Sav induced by Hpo. The mobility shift assay was employed. The loading volume was adjusted according to the total Sav protein level. (C) Par-1 disrupts the interaction of the Hpo-Sav complex. S2 cells were transfected with the indicated constructs followed by immunoprecipitation to test whether the interaction between Sav and endogenous Hpo was affected by Par-1. Note that less Sav interacted with Hpo in the presence of Par-1. (D–D″) Par-1 RNAi is incapable of inhibiting *sav* mutant-induced adult eye overgrowth. The clones were generated using the MARCM system. The genotypes are as following: *eyflp, ubi-Gal4, UAS-GFP; FRT82B/FRT82BGal80* (D), e*yflp, ubi-Gal4, UAS-GFP; FRT82B Sav^SH13^*/*FRT82B Gal80* (D′), and *eyflp, ubi-Gal4, UAS-GFP; Par-1-RNAi; FRT82B Sav^SH13^*/*FRT82B Gal80* (D″). (E) The proposed mechanism of Par-1 regulation of the Hpo pathway (see text for further detail).

Given that Par-1 functions upstream of *hpo* (Figure 4), we speculated that Par-1 modulated the function of the Hpo-Sav complex. To test our hypothesis, we first examined whether Par-1 bound to the Hpo-Sav complex using a co-immunoprecipitation assay. As expected, full-length Par-1 interacted with both Hpo and Sav, although these associations were weak (Figure 5A–5B). In addition, we also found that the HA-tagged N-terminal fragment of Par-1 (Par-1-N, Figure S4A) had a strong association with both Hpo and Sav, whereas there was only a weak interaction between the Par-1 C-terminal fragment (Par-1-C, Figure S4A) and Hpo/Sav had been detected (Figure 5A–5B). Importantly, the interaction between Par-1 and Hpo/Sav was specific because neither the full-length nor the N-terminal fragment of Par-1 co-immunoprecipitated with Merlin (unpublished data), Wts, or Mats (Figure S4B). On the basis of the previous results that Par-1 functioned in a kinase-dependent manner in Hpo signaling, we performed a phosphorylation shift experiment using a phos-tag gel to determine whether Par-1 affected the phosphorylation of Hpo and Sav *in vitro*. The Phos-Tag is a phosphate binding compound that, when incorporated into polyacrylamide gels, results in an exaggerated mobility shift for phosphorylated proteins, which is dependent upon the degree of phosphorylation [18],[55]. We observed that Flag-Hpo cotransfected with Par-1, but not Par-1-KD, in S2 cells that exhibited a mobility shift (Figure S5A). However, such a mobility shift was not detected for Sav when it was cotransfected with either Par-1 or Par-1-KD (Figure 6B, compare lanes 3–4 with lane 1). To remove the effect of Hpo auto-phosphorylation, a kinase-dead form of Hpo (Hpo-KD) was also tested in the phosphorylation shift experiment. As shown in Figure 5C, Par-1 also induced Hpo-KD to generate a phosphorylation mobility shift. These results suggested that Par-1 specifically induced Hpo phosphorylation in S2 cells.

To identify which sites on Hpo were affected by Par-1, we cotransfected Flag tagged Hpo with HA tagged Par-1 in S2 cells. Flag-Hpo protein was then immunoprecipitated and separated using SDS-PAGE and harvested for a semi-quantitative mass spectrometric (MS) analysis (details are described in the Materials and Methods). According to the MS results, we identified four potential phosphorylation sites that were affected by Par-1 expression, S30, S66, Y365, and T615. We then generated different Hpo variants by individually mutating these candidate sites and then examined how these mutations affected Par-1-induced Hpo phosphorylation. Interestingly, only Hpo (S30A) failed to generate mobility shifts in a direct Western blot analysis compared to wild-type Hpo protein (Figure S5C), while other Hpo mutations, T615A, S66A, and Y365E, did not affect the mobility shift upon Par-1 induction (Figure S5B–S5C). Moreover, we also found that Hpo (S30A)-KD could not be shifted by Par-1 (Figure 5D). These results suggested that Par-1 may regulate the phosphorylation of Hpo at Ser30. To verify our prediction, an antibody that specifically recognized phosphorylated Hpo at Ser30 was generated. As shown in Figures 5E and S5D, Par-1 but not Par-1-KD was able to induce Hpo phosphorylation at Ser30. To validate the phospho-Hpo S30 antibody, a phosphatase treatment was applied. Upon phosphatase treatment, the p-Hpo(S30) band was undetectable (Figure 5E, compare lane 5 with lane 2). To further test the specificity of this antibody, we transfected Hpo(S30) mutants with Par-1. As shown in Figure 5F and in Figure S5E, in the presence of Par-1, Hpo(S30A) could not be detected by the p-Hpo(Ser 30) antibody, whereas wild-type Hpo(S30) was detected. These findings indicated that Par-1 regulated the phosphorylation of Hpo at Ser30.

### Par-1-Induced Hpo Ser30 Phosphorylation Regulates Hpo Activity

In recent proteome-wide phosphorylation studies using *Drosophila* embryos [40], it was suggested that Hpo was phosphorylated at Ser30 *in vivo*, indicating an important role for the Ser30 site in the regulation of Hpo activity. To determine the biological significance of Hpo phosphorylation at Ser30 induced by Par-1, we first detected whether Ser30 phosphorylation state affects Hpo phosphorylation at Thr195, which was important for Hpo activation. As shown in Figure 5G–5H, Par-1, but not Par-1-KD, significantly inhibited Hpo phosphorylation levels at Thr195, whereas this inhibitory effect was abolished when the Ser30 site was mutated. More importantly, phosphorylation at Thr195 was slightly elevated when Ser30 was mutated into an alanine (Figure 5G, compare lane 4 with lane 1, and Figure 5H). These findings suggested that Par-1 regulated Hpo activity via antagonizing phosphorylation at the Thr195 site by regulating Ser30 phosphorylation. It has been reported that the Hpo Thr195 site is not only auto-phosphorylated but also phosphorylated by Tao-1 [37],[38], which is a partner of Par-1 in the regulation of microtubule dynamics. Thus, we asked whether Par-1-induced phosphorylation at Ser30 also affected Tao-1-mediated phosphorylation at Thr195. As shown in Figure S5F, consistent with the results shown in Figure 5G–5H, Par-1 suppressed Tao-1-mediated phosphorylation at Thr195. The antagonistic effect of Par-1 and Tao-1 on Hpo phosphorylation at Thr195 motivated the examination of the interrelationship of Par-1 and Tao-1 in the Hpo pathway. We found that Tao-1 disrupted Par-1-induced a phosphorylation mobility shift of Hpo-KD (Figure S5F), suggesting that the function of Par-1 in the Hpo pathway was modulated by upstream signaling.

Because Hpo(S30A) demonstrated a higher activity compared to the Hpo wild type, we examined whether Hpo(S30A) exerted its inhibitory effect on Yki. We found that Hpo(S30A) resulted in a dramatic Yki mobility shift, whereas the Hpo wild type resulted in a moderate phosphorylation of Yki (Figure 5I, compare lane 2 with lane 6). Furthermore, we found that co-transfection of the Hpo(S30A) mutant blocked Par-1-induced Yki dephosphorylation (Figure 5I, compare lane 3 with lane 7), which further confirmed our conclusion that Par-1 modulated Hpo activity by regulating Hpo phosphorylation at Ser30.

To further investigate the role of Par-1-induced Hpo phosphorylation at Ser30 in Hpo activity regulation, we generated transgenic flies of *attB-UAS-Hpo* variants at the 75B1 *attP* locus, which ensured equal expression of different forms of the Hpo mutant. Because the flies were grown at room temperature, overexpression of Hpo under the control of *GMR-Gal4*, *Ci-Gal4*, and *hh-Gal4* resulted in adult lethality; therefore, we selected weak drivers, such as *C765*, which induced gene expression moderately in the *Drosophila* wing to compare the activity of *Hpo* variants. Consistent with our *in vitro* studies, Hpo(S30A) mutants exhibited much smaller wings compared to wild-type Hpo flies (Figure 5J–5K), indicating that Hpo(S30A) variants demonstrated a higher activity than wild-type Hpo *in vivo*. To further strengthen this conclusion, we used an inducible *Ci-Gal4*, which was controlled by a temperature-sensitive Gal80, to drive Hpo expression in order to exclude the early lethality. Because some progeny were viable when Hpo expression was induced after pupa formation, we then compared the activity of Hpo variants by measuring the wing size of the survivors and calculated the mortality rate. We found that survivors with Hpo expression maintained a relatively normal wing size (Figure S5G–S5H), while survivors with Hpo(S30A) expression demonstrated much smaller wings compared to controls (Figure S5G–S5H). Meanwhile, Hpo(S30A) flies exhibited a higher mortality rate compared to wild-type Hpo flies (Figure S5I). These findings suggested that the activity of Hpo(S30A) mutants was higher compared to wild-type Hpo. Taken together, on the basis of the above biochemical and *in vivo* evidence, we speculated that Par-1 inhibited Hpo activity via the regulation of Hpo phosphorylation at the Ser30 site.

### Par-1 Inhibits Hpo-Sav Association in a Kinase-Dependent Manner

We observed that Par-1 blocked Hpo-induced Sav stabilization in a kinase-dependent manner (Figure S5C, and Figure 6A, compare lane 3 with lane 2). Thus, we examined whether Par-1 induced Sav destabilization, which was dependent on the phosphorylation of the Hpo Ser30 site. Interestingly, we found that the stabilization of Sav by Hpo was not affected by Par-1-induced Hpo phosphorylation at Ser30 because Hpo(S30A) mutants were still able to stabilize Sav, and this stabilization could be reversed by Par-1 (Figure S6A). In addition to the change in Sav protein levels, Par-1 also decreased the phosphorylation status of Sav, which was induced by Hpo in a kinase-dependent manner (Figure 6B, compare lanes 5–6 with lane 2). Given that full-length Par-1 weakly interacted with both Hpo and Sav (Figure 5A–5B), we then determined whether Par-1 affected the association between Hpo and Sav. As shown in Figure 6C, Par-1, but not Par-1-KD, impaired the association of endogenous Hpo and transfected Sav, suggesting that the interaction between Hpo and Sav was disrupted by Par-1 overexpression. To mimic the disruption of the Hpo-Sav complex, we ablated *sav* using the MARCM system. We found that the growth defect induced by Par-1 RNAi was incapable of inhibiting *sav* mutant-induced clone growth (Figure S6B–S6C) and adult eye overgrowth (Figure 6D-–6D″), suggesting that Par-1 functioned upstream of *sav*. These observations supported the notion that Par-1 kinase activity was important to restrain the function of the Hpo-Sav complex.

On the basis of the evidence provided above, we propose a model of how Par-1 restricts the activity of Hpo pathway (Figure 6E). Par-1 regulates Hpo phosphorylation at Ser30 to modulate the Hpo kinase activity; simultaneously, Par-1 also promotes the dissociation of Sav from Hpo, resulting in the dephosphorylation and destabilization of Sav, thereby repressing the function of the Hpo-Sav complex.

### The Mammalian Homologue of Par-1, MAP/MARK, Regulates the Hpo Pathway

**Figure 7:**
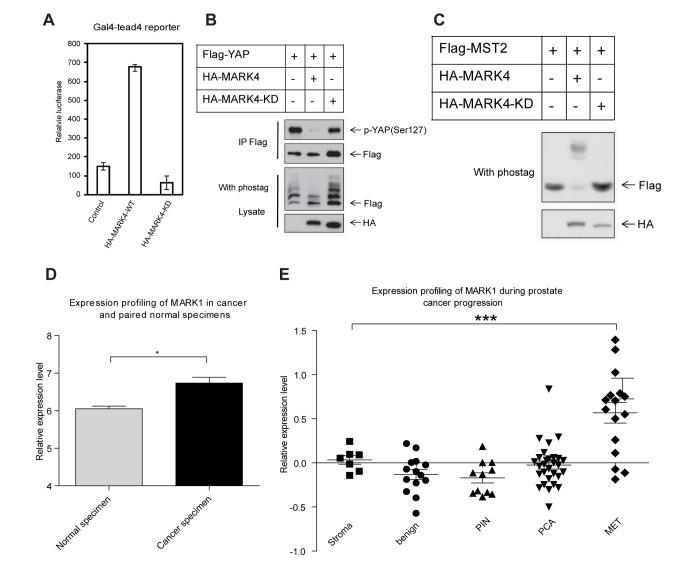
MARK, the mammalian homologue of Par-1, regulates the Hpo pathway. (A) MARK4 activates YAP transcriptional activity. The indicated plasmids were co-transfected with the *5XUAS-luc* reporter, *Gal4-TEAD4*, Flag-YAP, and CMV-β-galactosidase construct into HEK293T cells. Luciferase activity was measured and normalized against β-galactosidase activity. (B) Ectopic expression of MARK4 inhibits YAP phosphorylation. HEK293T cells were transfected with the indicated constructs, and YAP was immunoprecipitated using the anti-Flag antibody. YAP phosphorylation was detected using Western blot analysis with a phospho-YAP specific antibody and determining its mobility on a Phos-tag-containing SDS-PAGE gel. (C) MARK4 induces the phosphorylation of MST2. HEK293T cells were transfected with the indicated constructs, and MST2 phosphorylation was analyzed by electrophoresis on a Phos-tag-containing gel and through Western blotting with an anti-Flag antibody. (D) MARK1 expression is significantly upregulated in squamous lung cancer biopsy specimens compared with matched control specimens. The expression profiling data were downloaded from the GEO dataset GDS1312. These data were expressed as the mean ± SEM and were analyzed using the paired *t*-test. *p<0.05. (E) Prostate cancer progression is accompanied by an increased expression of MARK1. PIN, prostatic intraepithelial neoplasia; PCA, localized prostate cancer; MET, metastatic prostate cancer. The expression data were obtained from GEO dataset GDS3289. All of the results were expressed as the mean ± SEM. ***p<0.001.

An evolutionally conserved function of Par-1 in regulating microtubule dynamics has been reported [41]. To determine whether the function of Par-1 on the Hpo pathway is conserved, the human homologue of Par-1, MARK1 and MARK4 were cloned. The Gal4-tead4 reporter [56] was used to examine the effect of MARK1 and MARK4 on the mammalian Hpo pathway. As expected, both MARK1 and MARK4, but not the kinase-dead form of MARK4, activated the YAP transcription co-activator activity (Figures 7A and S7A). Since MARK4 activated YAP more than MARK1, we investigated whether MARK4 affected YAP phosphorylation. Indeed, the phosphorylation levels of YAP were significantly decreased upon MARK4 overexpression (Figure 7B), indicating that MARK inhibited Hpo signaling in mammals. Finally, we investigated whether MARK also resulted in MST (human homologue of Hpo) phosphorylation. We found that both MARK4 and MARK1 induced mobility shifts of MST2 (Figures 7C and S7B). Taken together, these findings suggested that the inhibitory function of Par-1 on Hpo signaling was conserved from *Drosophila* to mammals. Our study suggested that Par-1 may have a procarcinogenic role because its hyperactivation in *Drosophila* is sufficient to induce tissue overgrowth, and in mammals, Par-1 is sufficient to activate YAP. Interestingly, MARK4 has been suggested to play a role in hepatocellular carcinogenesis and gliomagenesis [51],[52]. However, whether MARK1 is also involved in carcinogenesis is largely unknown. To further elucidate this question, we characterized the mutation and expression of MARK1 in different cancer samples using the COSMIC and GEO databases. Although no mutation or deletion of MARK1 has been reported in the tumors that were surveyed, the transcription levels of MARK1 showed significant upregulation in squamous lung cancer samples and during the progression of prostate cancer (Figure 7D–7E). Taken together, these findings suggested that Par-1 was a potential oncogene and that its regulatory role in Hpo signaling could be conserved.

## Discussion

The Hpo signaling pathway has emerged as a conserved pathway that controls tissue growth and balances tissue homeostasis via the regulation of the downstream Sd-Yki transcription complex. Despite the importance of this pathway in development and carcinogenesis [2],^6^, many unknown regulators of the Hpo pathway remain to be identified. Here, we identified Par-1 as one such Hpo pathway regulator via a genetic overexpression screen using *Drosophila* EP lines. In this study, we demonstrated that Par-1 was essential for the restriction of Hpo signaling. We also demonstrated that overexpression of Par-1 promoted tissue growth via the inhibition of the Hpo pathway, whereas loss of Par-1 promoted Hpo signaling to suppress growth and induce apoptosis. Using the *Drosophila* eye and wing imaginal discs as well as cultured cells, we provide the first genetic and biochemical evidence for a function of Par-1 in the Hpo pathway.

Although the conserved function of Hpo has been well studied, the regulatory mechanism of its kinase activity is still largely obscure. Currently, the regulatory mechanism of Hpo kinase activity was believed to mainly be dependent on autophosphorylation by altering the phosphorylation status of the Thr195 site [37],[53],[57]. However, whether the uncharacterized phosphorylation events of Hpo, which have been identified in several recent proteome-wide phosphorylation studies [39],[40], contributed to the regulation of Hpo activity is still unknown. By studying the mechanism underlying Par-1 function in Hpo signaling, we demonstrated that Par-1 induced Hpo phosphorylation at Ser30 and this lead to the regulation of Hpo kinase activity.

Although we have extensively studied how Par-1 regulates the Hpo pathway in this study, several unresolved questions remain. The interaction between Par-1 and Hpo/Sav may be tightly regulated because full-length Par-1 only weakly interacted with Hpo/Sav, unlike the interaction with the N-terminal fragment of Par-1 (Figure 5A–5B). However, the triggering signal for Par-1 to interact with Hpo/Sav is still unknown. It has been reported that Par-1 was activated by Tao-1 and LKB1 [49],[50]. In this study, we established that Par-1 antagonized Tao-1 in Hpo signaling, and interestingly, in *Drosophila*, the antagonistic relationship between Par-1 and Tao-1 in microtubule regulation has been previously reported [57]–[59]. Thus, it is unlikely that Tao-1 functions as the trigger. We then investigated whether LKB1 functioned as an activator of Par-1 in Hpo signaling by expressing the LKB1 transgene in different organs. Unlike Par-1, ectopic LKB1 expression limited both wing and eye growth (unpublished data), indicating that LKB1 was also not the trigger.

We have shown that Par-1 and Tao-1 exhibited opposing effects on Hpo signaling (Figure S5F). Given that Tao-1 and Par-1 were partners that regulated microtubule dynamics via the phosphorylation of Tau [48], Tau may have a function in Hpo signaling. To investigate this hypothesis, we employed genetic and biochemical studies and found that Tau RNAi failed to suppress the expression of Hpo pathway-responsive genes (Figure S8A–S8B). In addition, Tau did not trigger Hpo phosphorylation and Sav dissociation *in vitro* (Figure S8C–S8D), indicating that Par-1 regulated Hpo signaling independent of Tau. Interestingly, it has been previously suggested that Par-1 did not regulate Tau activity in *Drosophila* [46], indicating an evolutionary difference between Par-1 and Tau-1 function.

We have provided evidence that Par-1 regulated Hpo signaling via the phosphorylation of Hpo or the destruction of the Hpo/Sav complex. Because Par-1 is a well-known polarity regulator and polarity components, such as Crumb and Lgl, have been shown to be involved in the Hpo signaling pathway [35], it is possible that Par-1 may regulate Hpo signaling via a polarity complex, or its activity might be regulated via a polarity complex. Indeed, the localization of Crumb and Patj were affected by Par-1 expression (unpublished data). Thus, further studies on polarity complexes and Hpo signaling will help elucidate this problem.

## Materials and Methods

### Cloning, Mutants, Transgenes, and *Drosophila* Genetics

Par-1 fragments were amplified from the BDGP DGC Clone (number RE47050) using the PCR. The sequence of full-length Par-1 used in this study was the same as Par-1-N1S (accession number NP_001014542). which has been previously described [43]. The Par-1 kinase-dead mutant was generated by converting the conserved Ser412 and Thr408 into alanine at the activation loop of the Par-1 catalytic domain. All of the primers used in this study are available upon request.

The EP library for the screen was a gift from Jianming Chen (Third Institute of Oceanography, State Oceanic Administration, China). *Par-1^w3^* is a null allele from the St Johnston lab, and FRT/FLP-mediated mitotic recombination was used to generate mutant clones, as previously described [20]. The genotypes used to generate the clones were the following: *FRTG13 Par-1^w3^/eyflp; FRTG13 ubi-GFP*. Par-1 RNAi fly was purchased from the Bloomington *Drosophila* Stock Center (stock number 32410). To generate the Par-1 RNAi-2 transgenic fly, an artificial microRNA method was adopted, which was reported to efficiently silence gene expression [60]. Briefly, we designed two hairpin oligos that were targeted to the 1227–1247 base pair and the 1551–1571 base pair regions of Par-1-N1S (accession number NM_001014542). Next, these two hairpin oligos were ligated to create a tandem hairpin RNA. The procedure for this complete construction can be found in the supplementary information of Wang et al.'s study [61]. The following transgenes were used in this study: *bantam-lacZ* (a gift from Wei Du, The University of Chicago), *UAS-ex-RNAi* (V22994, VDRC), *UAS-fat-RNAi* (V9396, VDRC), and *UAS-Tau-RNAi* (V25024, VDRC). Other stocks included: *bantam sensor mic32-GFP, ex-lacZ, diap1-GFP3.5, hh-Gal4, GMR-Gal4, MS1096, act* > *CD2* > *Gal4, eyless-Gal4, diap1-lacZ, UAS-Yki, UAS-Yki-RNAi, UAS-Sd-RNAi, hpo^BF33^*, *wts^latsX1^,* and *Sav^SH13^*, which have all been previously described [20],[53]. The generation of the transgenes at the *attP* locus has been previously described [20]. Unless otherwise indicated, all of the flies were cultured at 25°C.

### Cell Culture, Transfection, Immunoprecipitation, Western Blot Analysis, Immunostaining, and the Luciferase Reporter Assay

S2 cells were maintained in *Drosophila* Schneider's Medium (Invitrogen) supplemented with 10% heat-inactivated fetal bovine serum, 100 U/ml of penicillin, and 100 mg/ml of streptomycin. The cells were incubated at 25°C in a humidified air atmosphere.

Plasmid transfection was performed using Lipofectamine (Invitrogen), according to the manufacturer's instructions. For all of the transfection experiments, a *ubiquitin-Gal4* construct was co-transfected with the *pUAST* expression vectors.

The procedures for the immunoprecipitation, Western blotting, and immunostaining analyses were previously described [20],[53]. The following antibodies were used in the immunoprecipitation or Western blot analyses: rabbit anti-Hpo antibody [53], rabbit anti-Phospho Hpo (Thr195) antibody (Cell Signaling Technology), mouse anti-Flag antibody (Sigma), mouse anti-Myc antibody (Santa Cruz), mouse anti-V5 antibody (Invitrogen), and mouse anti-GFP/CFP antibody (Santa Cruz). The antibodies used in the immunostaining experiments were the following: rabbit anti-Par-1 antibody (a gift from the Montell lab, Lerner Research Institute), mouse anti-DIAP1 (a gift from Bruce A. Hay, California Institute of Technology), rat anti-cubitus interruptus (Ci) antibody (Developmental Studies Hybridoma Bank, DSHB), rabbit anti-lacZ antibody (Invitrogen), mouse anti-CD2 antibody (Invitrogen), and rabbit anti-cleaved caspase-3 antibody (Cell Signaling Technology). The rabbit anti-Phospho Hpo(Ser30) antibody was generated by Abgent.

For the luciferase reporter assay, the *3×Sd2-Luc* reporter has been previously described [20]. The luciferase assay was performed using the Dual Luciferase Assay System (Promega).

### Phosphorylation Mobility Shift Assay

For the phosphorylation mobility shift assays, Phos-Tag AAL-107 (FMS Laboratory) was introduced to enlarge the mobility shift. The operating procedure was performed according to the manufacturer's instructions. For all of the mobility shift assays, the protein samples were processed using an SDS-PAGE gel under a low voltage. According to the molecular weight of the protein, a 6% or 8% resolving gel was used for the Wts and the Sav and Hpo mobility shift assays, respectively.

### *In Vitro* Kinase Assay

Immunoprecipitated cell lysates or purified protein were directly incubated in 20–40 µl of kinase assay buffer (250 mM HEPES, pH 7.4, 0.2 mM EDTA, 1% glycerol, 150 mM NaCl, and 10 mM MgCl_2_). The reaction were initiated by the addition of an ATP mixture (2 µl 1 mM ATP, 0.2 µl [γ-^32^P] ATP [10 mCi/ml]) and then incubated at 30°C for 30 min. The reactions were terminated by the addition of an SDS sample buffer. Next, the samples were boiled for 5 min at 100°C followed by SDS-PAGE and autoradiography.

### Mapping Phosphorylation Sites by Mass Spectrometry

S2 cells were transfected with Flag-Hpo or cotransfected with Flag-Hpo and Par-1. 48 h after transfection, the cells were harvested and then lysed. SDS-PAGE and Colloidal Blue staining (Invitrogen, LC6025) were then performed on the protein samples. The Hpo protein was cut from the gel and sent to the Protein Center, SIBCB for mass spectrometric analysis. A detailed procedure of the mass spectrometric analysis may be obtained from the Protein Center, SIBCB. The candidate sites were identified by the increased phosphorylation abundance in the cotransfected Flag-Hpo and Par-1 cells versus Flag-Hpo transfected cells.

### Statistical Analysis

All of the data in this study were expressed as the mean ± standard error of the mean (SEM) and were analyzed using Student's *t* test by R 2.9.0. The results were considered statistically significant if *p*<0.05.

## Supporting Information

Figure S1 (A–A′) *Drosophila* wings of wild type (A) or wings expressing Par-1 (A′) with *hh-Gal4*. The posterior compartments were indicated by a pseudo-gray color. (B) Quantification of the relative P-compartment area of the wings. The results were calculated as the area of the P-compartment divided by the entire wing area. The results represented the mean ± SEM. * mean *p*<0.05 (*n*>6) for each genotype. (C–C″) Overexpression of Par-1 promotes the transcription of *diap1*. Cells expressing *UAS-Par-1* were labeled by the lack of CD2 expression (indicated arrows). Note the upregulation of *diap1* transcription via ectopic Par-1 expression. (D) Inability of Par1-KD to autophosphorylate. Myc-tagged Par-1 or Par-1-KD was immunoprecipitated and subjected to an *in vitro* kinase assay. (E–F) Western blot analysis of extracts from third-instar larval eye discs (E) and wing discs (F) to show the expression level of Par-1 and Par-1-KD.
(TIF)

Figure S2 (A–C′) Wild-type wing discs (A–A′) or wing discs expressing Par-1 RNAi (B–B′) or Par-1 RNAi-2 (C–C′) with *hh-Gal4* were immunostained with anti-Ci (red) and anti-Par-1 (green) to detect RNAi efficiency. The arrows indicate the P-compartment. Both RNAi lines could efficiently knock down the endogenous Par-1 protein. (D–D′) Adult wings of wild type (D) or wings expressing Par-1-RNAi-2 (D′) with *MS1096*. Note the reduced organ size induced by Par-1 RNAi-2. (E–G′) Wing discs expressing Par-1 RNAi-2 in the P-compartment with *hh-Gal4* were immunostained to demonstrate the expression of *ex-LacZ* (E–E′), *diap1- GFP3.5* (F–F″), and bantam sensor *mic32-GFP* (G–G″). Note that Par-1 RNAi-2 downregulated the expression of Hpo-responsive genes. The arrows indicate the P-compartment.
(TIF)

Figure S3 (A–B′) Gain-of-function of the Par-1-induced phenotype is blocked by Yki RNAi. UAS-2*Myc-Par-1 (A–A′), UAS-2*Myc-Par-1; *UAS-Yki RNAi* (B–B′) were expressed under the control of *act*>*CD2*>*Gal4* to detect changes in *diap1-LacZ*. Cells expressing the indicated transgenes were marked by Myc tag (indicated by arrows). Note that the upregulation of *diap1-LacZ* induced by ectopic Par-1 was completely suppressed by Yki RNAi. (C–F′) Par-1 is functionally dependent on Sd in the Hpo pathway. Adult wings expressing UAS-2*Myc Par-1; *UAS-Sd RNAi* (C′) showed a similar phenotype as that of Sd RNAi (C). Furthermore, wild-type wing discs (D–D′), wing discs expressing UAS-2*Myc-Par-1 (E–E′) or *UAS-Myc-Par-1*; *UAS-Sd RNAi* (F–F′) in the P-compartment were immunostained to demonstrate the expression of *diap-GFP3.5*. The P-compartment was marked by the loss of Ci or Myc tag (red) and is indicated by arrows. Note that coexpression of Sd RNAi reversed the upregulation of *diap1-GFP 3.5* induced by Par-1. (G–G′″) *Drosophila* wings of the indicated genotypes are shown. Note that the enlarged wing size induced by *ex* RNAi was reversed by Par-1 RNAi. (H–H′″) *Drosophila* wings of the indicated genotypes are shown. Note that Par-1 RNAi reduced wing size even in the fat-RNAi condition.
(TIF)

Figure S4 (A) A schematic representation of the Par-1 full-length structure or its truncated forms. (B) Immunoprecipitation between Par-1-N, Wts and Mats. S2 cells were transfected with the indicated constructs followed by co-immunoprecipitation. Note that Wts and Mats were unable to interact with the N-terminal of Par-1.
(TIF)

Figure S5 (A) Par-1 regulates Hpo phosphorylation *in vitro*. S2 cells were transfected with the indicated constructs. Cell lysates were subjected to the phosphorylation mobility shift assay. Note the phosphorylation shift of Hpo in the presence of Par-1 but not with Par-1 KD. (B–C) Hpo(S30A) mutants blocked the Par-1-induced Hpo phosphorylation shift but Hpo (T615A), Hpo(S66A) and Hpo(Y365E) did not. (D–E) Par-1 induces Ser30 phosphorylation of Hpo in S2 cells. S2 cells were transfected with the indicated constructs. The cell lysates were subjected to a Western blot analysis. Note that the phospho Hpo(Ser30) antibody could only detect Hpo but not Hpo(S30A) phosphorylation induced by Par-1. (F) Par-1 and Tao-1 antagonization regulates Hpo phosphorylation. S2 cells were transfected with the indicated plasmids, and the cell lysates were subjected to a direct Western blot analysis or a phosphorylation mobility shift assay. Note that Tao-1 partially inhibited the Par-1-induced Hpo phosphorylation mobility shift, while Par-1 inhibited Tao-1-induced Hpo Thr195 phosphorylation. (G–I) Hpo(S30A) showed enhanced activity compared with wild-type Hpo *in vivo*. Adult wings of wild-type (G), wings expressing Hpo (G′), and wings expressing Hpo(S30A) (G″) under the control of *tub-Gal80^ts^; Ci-Gal4*. Note that the Hpo Ser30 mutant induced smaller wings (H) and a higher mortality rate (I) compared to wild-type Hpo. The relative wing size (H) was quantified using an unpaired *t*-test. The results represented the mean ± SEM. **p<0.01, (*n*>6) for each genotype. The percentage of lethal flies (I) was calculated by dividing the number of lethal pupas by the total number of pupas. To induce Hpo expression, fly progeny were transferred to a 29°C incubator at different developmental stages.
(TIF)

Figure S6 (A) Par-1 destabilizes Hpo-induced Sav accumulation independent of Par-1 phosphorylation at the Hpo Ser30 site. S2 cells were transfected with the indicated constructs followed by a Western blot analysis. Note that both wild-type Hpo and Hpo(S30A) could stabilize Sav. In addition, stabilized Sav can be destabilized by Par-1 overexpression. (B) *par-1* functions upstream of *sav* in the Hpo pathway. Clones were generated using the MARCM system. The following genotypes were used: *ey-flp, Ubi-Gal4, UAS-GFP; FRT82B Sav^SH13^*/*FRT82B Gal80* (left panel), and *eyflp, ubiGal4, UAS-GFP; Par-1-RNAi; FRT82B Sav^SH13^*/*FRT82B Gal80* (right panel). (C) Quantification of the relative clone size. The relative clone size was calculated as the GFP area divided by the entire disc area. All of these data were expressed as the mean ± SEM. **p<0.01. **p<0.001. *n*>5, for each group.
(TIF)

Figure S7 (A) MARK1 significantly enhances the transcriptional activity of Yap. HEK293T cells were transfected with the indicated plasmids, and the cell lysates were directly subjected to a dual luciferase reporter assay. Note that MARK1 synergized with Yap to promote the transcriptional activity of TEAD. (B) MARK1 induces a MST2 phosphorylation mobility shift. HEK293T cells were transfected with the indicated plasmids and cell lysates were directly subjected to a phosphorylation mobility shift assay. Note that MST2 showed a shift band by MARK1 coexpression.
(TIF)

Figure S8 (A–B′) Wing discs expressing Tau RNAi (A–B′) with *hh-Gal4* were immunostained with anti-Ci (red) and anti-DIAP1(green A–A′) to demonstrate the expression level of DIAP1 (A–A′) and *mic32-GFP* (B–B′). Note that the expression of Tau RNAi failed to affect Hpo pathway-responsive gene expression. The arrows indicate the P-compartment. (C–D) S2 cells were transfected with the indicated constructs followed by Western blot analyses. Note that Tau affects neither the phosphorylation status of Sav (C) nor the mobility shift of Hpo (D).
(TIF)

## Abbreviations

Hpo: Hippo
Sav: Salvador
Yki: Yorkie
Mats: Mob as tumor suppressor
Sd: Scalloped
Ex: Expanded
MARK: microtubule affinity regulating kinase
KD: kinase dead
MARCM: mosaic analysis with a repressible cell marker
SEM: standard error of the mean
