# Supplementary figures and images for: Par-1 Regulates Tissue Growth by Influencing Hippo Phosphorylation Status and Hippo-Salvador Association
Source: PLoS Biol. 2013 Aug 6;11(8):e1001620. doi: 10.1371/journal.pbio.1001620 (PMC3735459; doi:10.1371/journal.pbio.1001620)

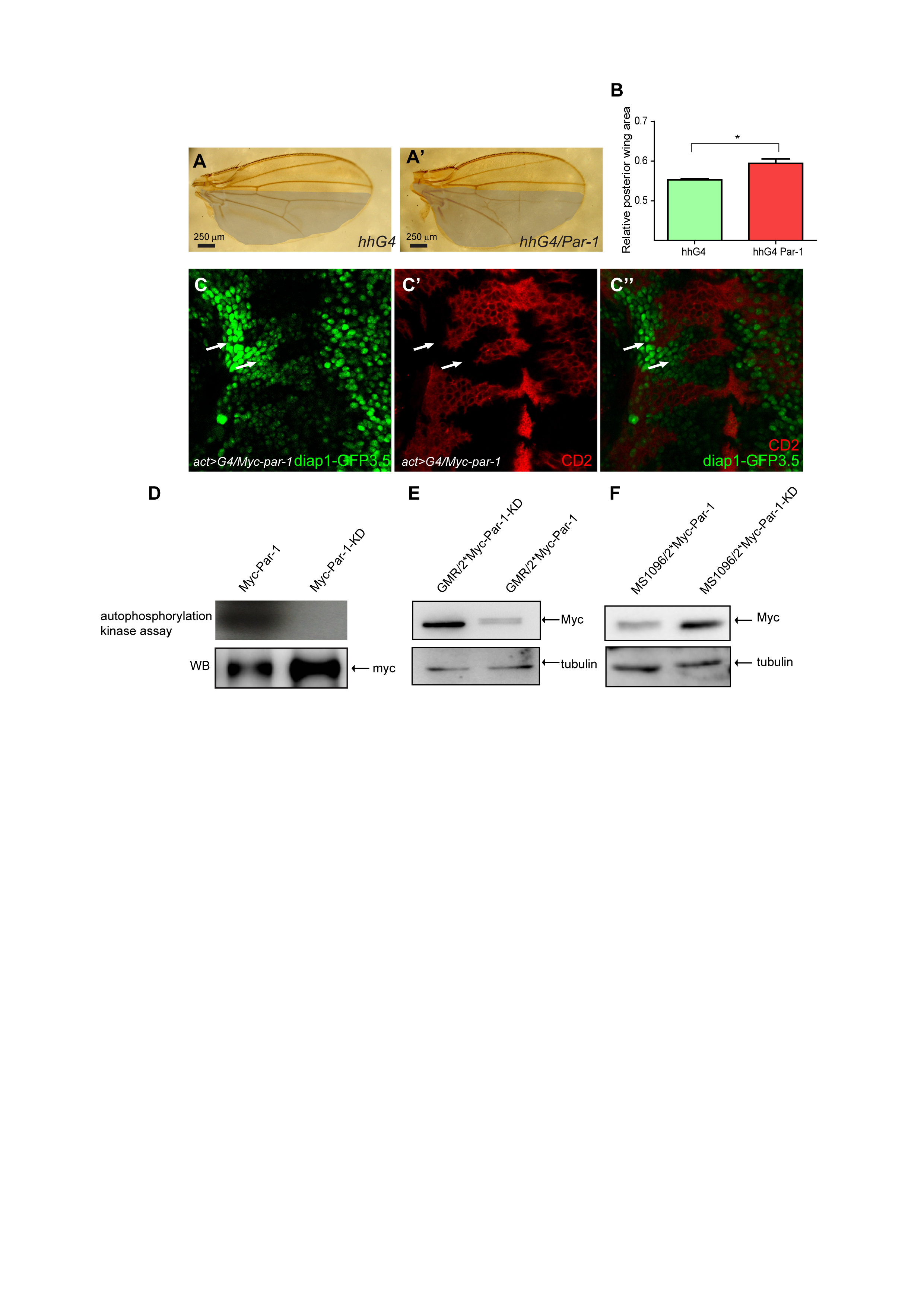

Supplement: Figure S1 — (A–A′) Drosophila wings of wild type (A) or wings expressing Par-1 (A′) with hh-Gal4. The posterior compartments were indicated by a pseudo-gray color. (B) Quantification of the relative P-compartment area of the wings. The results were calculated as the area of the P-compartment divided by the entire wing area. The results represented the mean ± SEM. * mean p<0.05 (n>6) for each genotype. (C–C″) Overexpression of Par-1 promotes the transcription of diap1. Cells expressing UAS-Par-1 were labeled by the lack of CD2 expression (indicated arrows). Note the upregulation of diap1 transcription via ectopic Par-1 expression. (D) Inability of Par1-KD to autophosphorylate. Myc-tagged Par-1 or Par-1-KD was immunoprecipitated and subjected to an in vitro kinase assay. (E–F) Western blot analysis of extracts from third-instar larval eye discs (E) and wing discs (F) to show the expression level of Par-1 and Par-1-KD. (TIF) [file pbio.1001620.s001.tif]

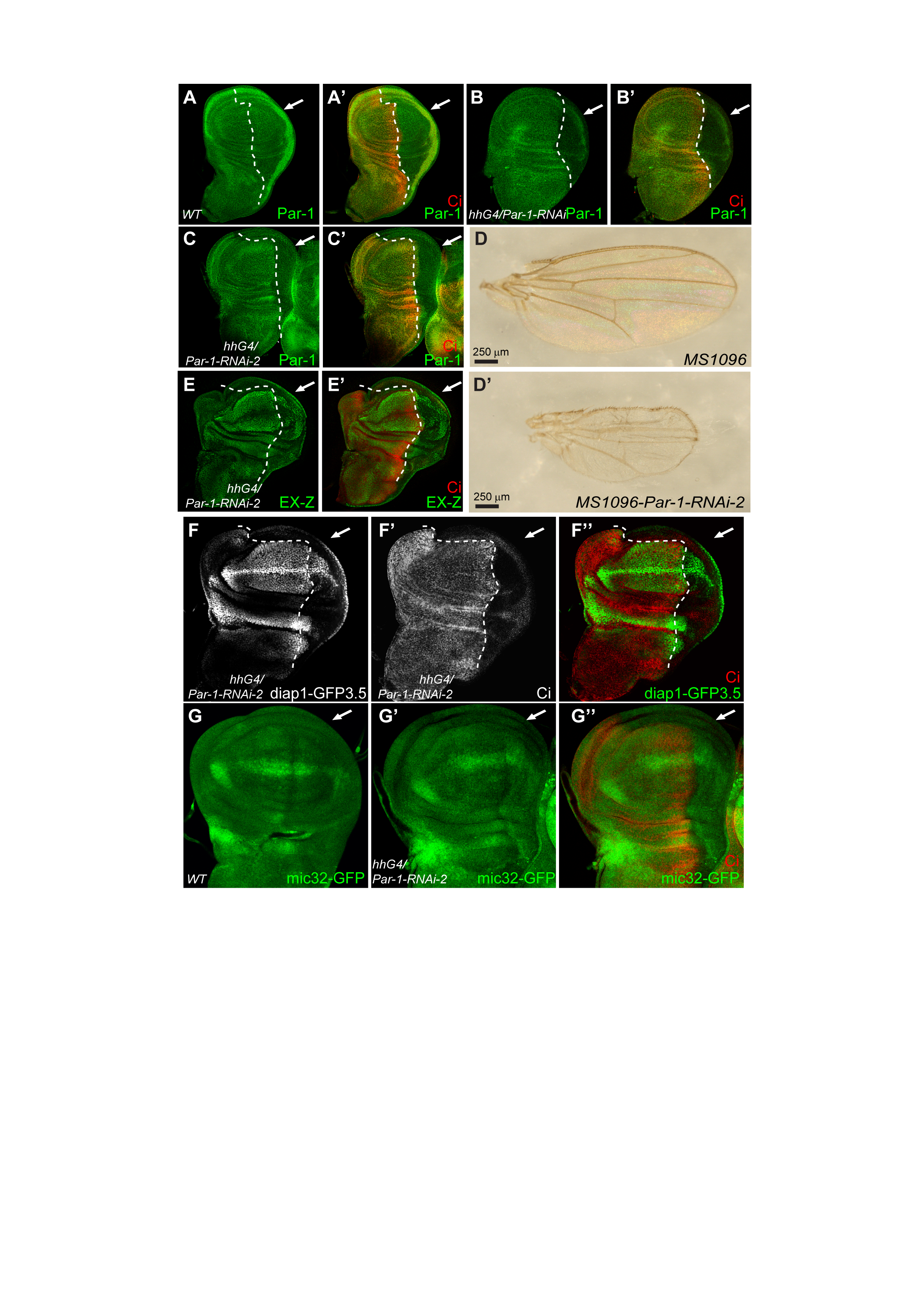

Supplement: Figure S2 — (A–C′) Wild-type wing discs (A–A′) or wing discs expressing Par-1 RNAi (B–B′) or Par-1 RNAi-2 (C–C′) with hh-Gal4 were immunostained with anti-Ci (red) and anti-Par-1 (green) to detect RNAi efficiency. The arrows indicate the P-compartment. Both RNAi lines could efficiently knock down the endogenous Par-1 protein. (D–D′) Adult wings of wild type (D) or wings expressing Par-1-RNAi-2 (D′) with MS1096. Note the reduced organ size induced by Par-1 RNAi-2. (E–G′) Wing discs expressing Par-1 RNAi-2 in the P-compartment with hh-Gal4 were immunostained to demonstrate the expression of ex-LacZ (E–E′), diap1- GFP3.5 (F–F″), and bantam sensor mic32-GFP (G–G″). Note that Par-1 RNAi-2 downregulated the expression of Hpo-responsive genes. The arrows indicate the P-compartment. (TIF) [file pbio.1001620.s002.tif]

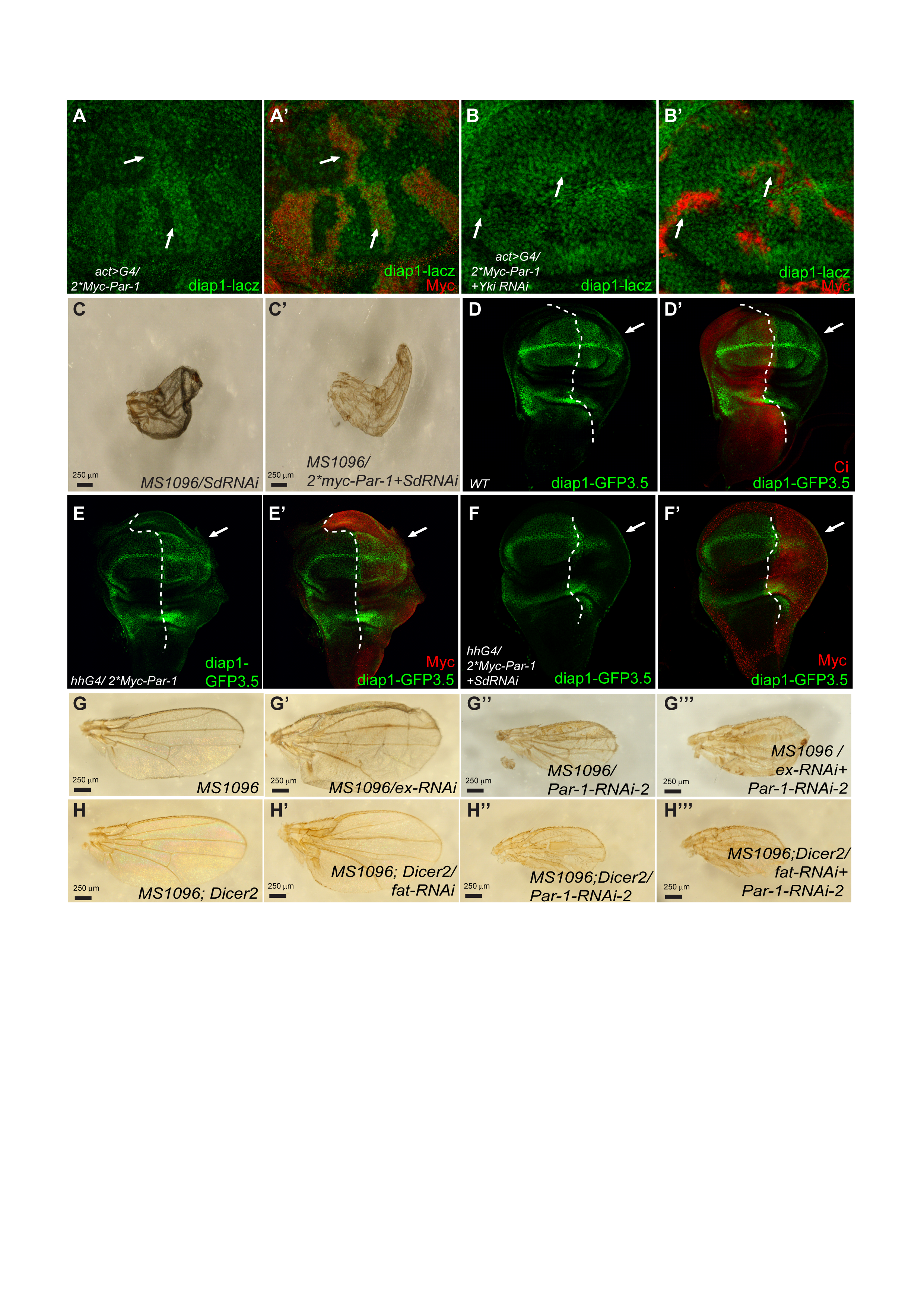

Supplement: Figure S3 — (A–B′) Gain-of-function of the Par-1-induced phenotype is blocked by Yki RNAi. UAS-2*Myc-Par-1 (A–A′), UAS-2*Myc-Par-1; UAS-Yki RNAi (B–B′) were expressed under the control of act>CD2>Gal4 to detect changes in diap1-LacZ. Cells expressing the indicated transgenes were marked by Myc tag (indicated by arrows). Note that the upregulation of diap1-LacZ induced by ectopic Par-1 was completely suppressed by Yki RNAi. (C–F′) Par-1 is functionally dependent on Sd in the Hpo pathway. Adult wings expressing UAS-2*Myc Par-1; UAS-Sd RNAi (C′) showed a similar phenotype as that of Sd RNAi (C). Furthermore, wild-type wing discs (D–D′), wing discs expressing UAS-2*Myc-Par-1 (E–E′) or UAS-Myc-Par-1; UAS-Sd RNAi (F–F′) in the P-compartment were immunostained to demonstrate the expression of diap-GFP3.5. The P-compartment was marked by the loss of Ci or Myc tag (red) and is indicated by arrows. Note that coexpression of Sd RNAi reversed the upregulation of diap1-GFP 3.5 induced by Par-1. (G–G′″) Drosophila wings of the indicated genotypes are shown. Note that the enlarged wing size induced by ex RNAi was reversed by Par-1 RNAi. (H–H′″) Drosophila wings of the indicated genotypes are shown. Note that Par-1 RNAi reduced wing size even in the fat-RNAi condition. (TIF) [file pbio.1001620.s003.tif]

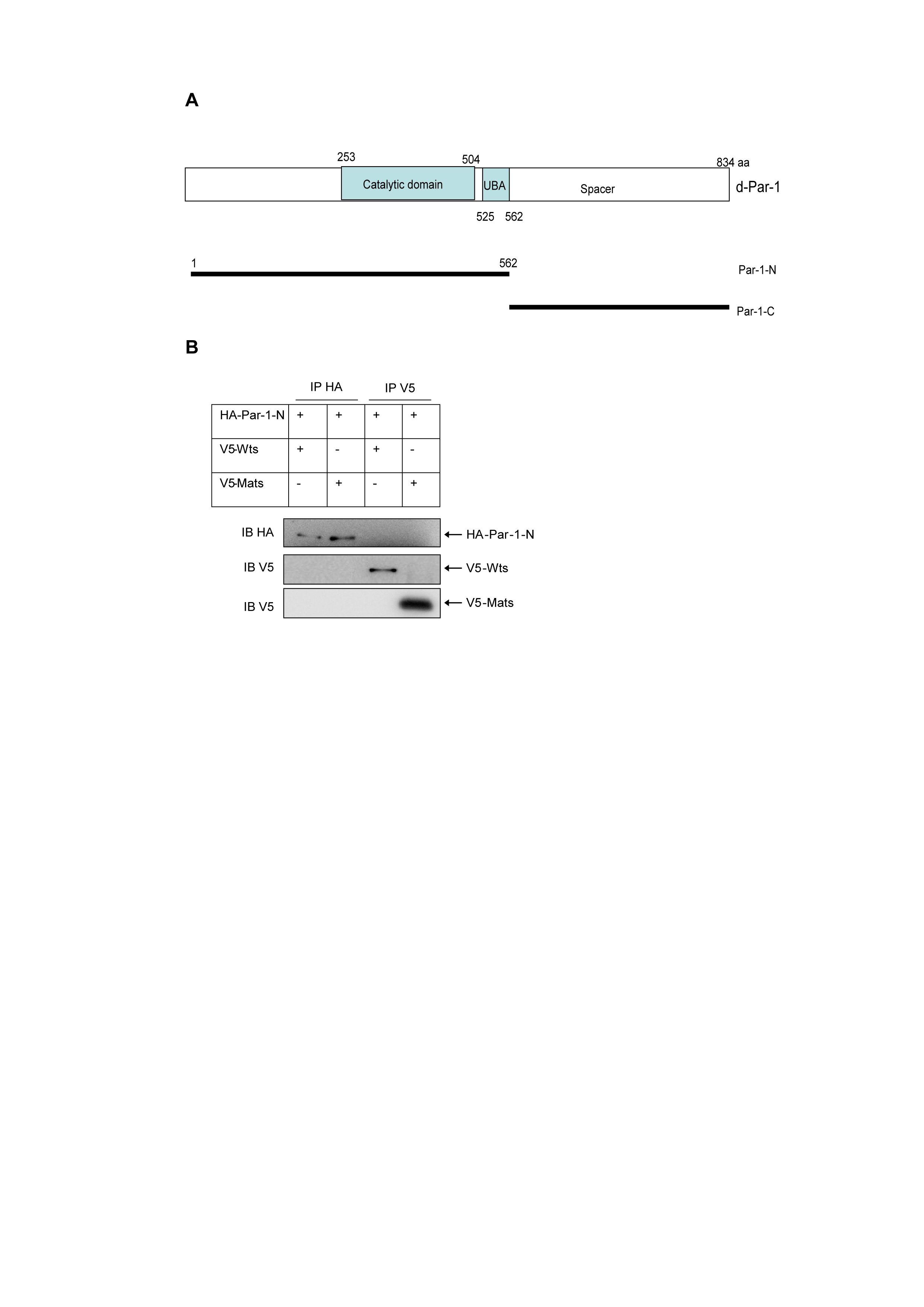

Supplement: Figure S4 — (A) A schematic representation of the Par-1 full-length structure or its truncated forms. (B) Immunoprecipitation between Par-1-N, Wts and Mats. S2 cells were transfected with the indicated constructs followed by co-immunoprecipitation. Note that Wts and Mats were unable to interact with the N-terminal of Par-1. (TIF) [file pbio.1001620.s004.tif]

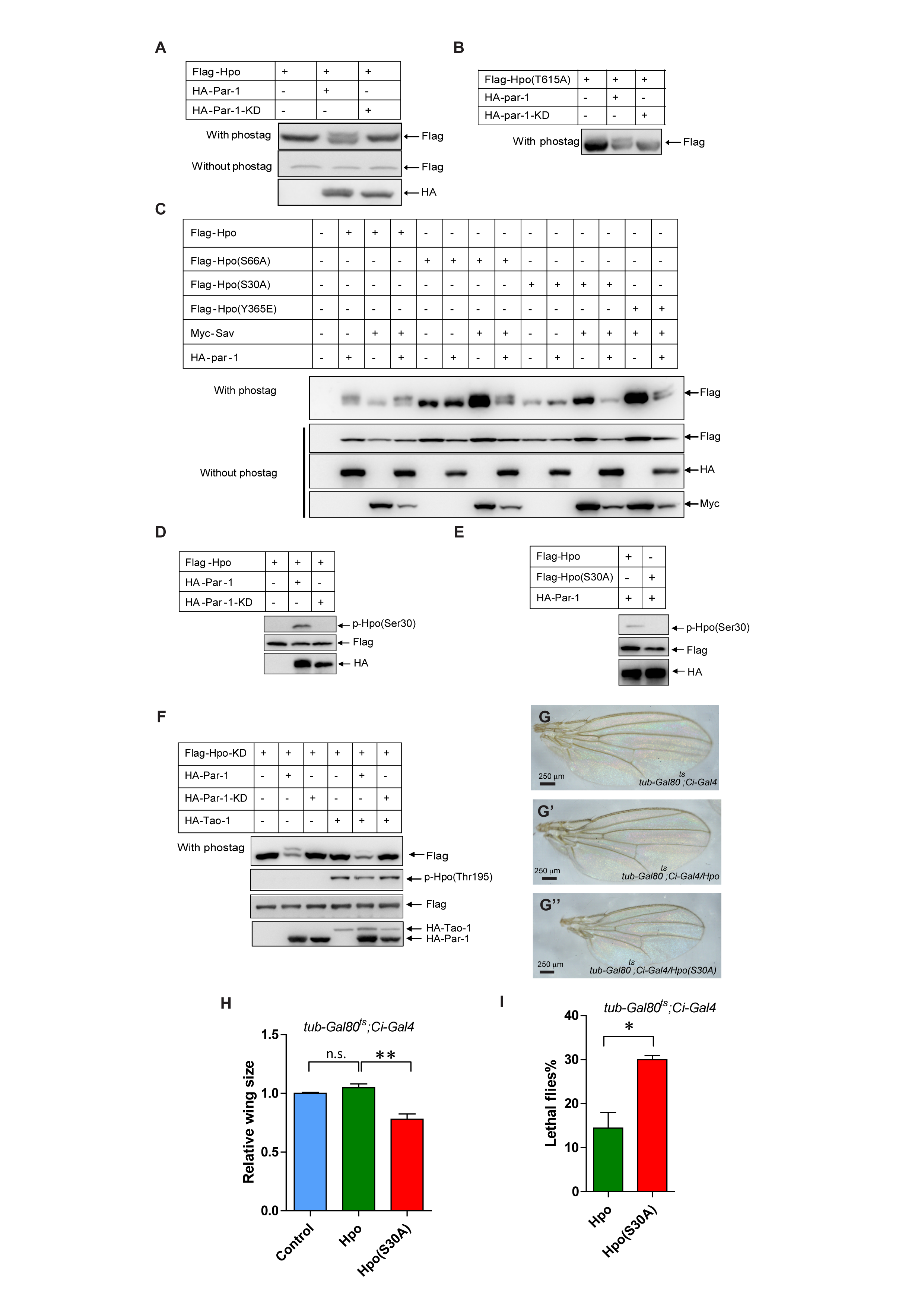

Supplement: Figure S5 — (A) Par-1 regulates Hpo phosphorylation in vitro. S2 cells were transfected with the indicated constructs. Cell lysates were subjected to the phosphorylation mobility shift assay. Note the phosphorylation shift of Hpo in the presence of Par-1 but not with Par-1 KD. (B–C) Hpo(S30A) mutants blocked the Par-1-induced Hpo phosphorylation shift but Hpo (T615A), Hpo(S66A) and Hpo(Y365E) did not. (D–E) Par-1 induces Ser30 phosphorylation of Hpo in S2 cells. S2 cells were transfected with the indicated constructs. The cell lysates were subjected to a Western blot analysis. Note that the phospho Hpo(Ser30) antibody could only detect Hpo but not Hpo(S30A) phosphorylation induced by Par-1. (F) Par-1 and Tao-1 antagonization regulates Hpo phosphorylation. S2 cells were transfected with the indicated plasmids, and the cell lysates were subjected to a direct Western blot analysis or a phosphorylation mobility shift assay. Note that Tao-1 partially inhibited the Par-1-induced Hpo phosphorylation mobility shift, while Par-1 inhibited Tao-1-induced Hpo Thr195 phosphorylation. (G–I) Hpo(S30A) showed enhanced activity compared with wild-type Hpo in vivo. Adult wings of wild-type (G), wings expressing Hpo (G′), and wings expressing Hpo(S30A) (G″) under the control of tub-Gal80ts; Ci-Gal4. Note that the Hpo Ser30 mutant induced smaller wings (H) and a higher mortality rate (I) compared to wild-type Hpo. The relative wing size (H) was quantified using an unpaired t-test. The results represented the mean ± SEM. **p<0.01, (n>6) for each genotype. The percentage of lethal flies (I) was calculated by dividing the number of lethal pupas by the total number of pupas. To induce Hpo expression, fly progeny were transferred to a 29°C incubator at different developmental stages. (TIF) [file pbio.1001620.s005.tif]

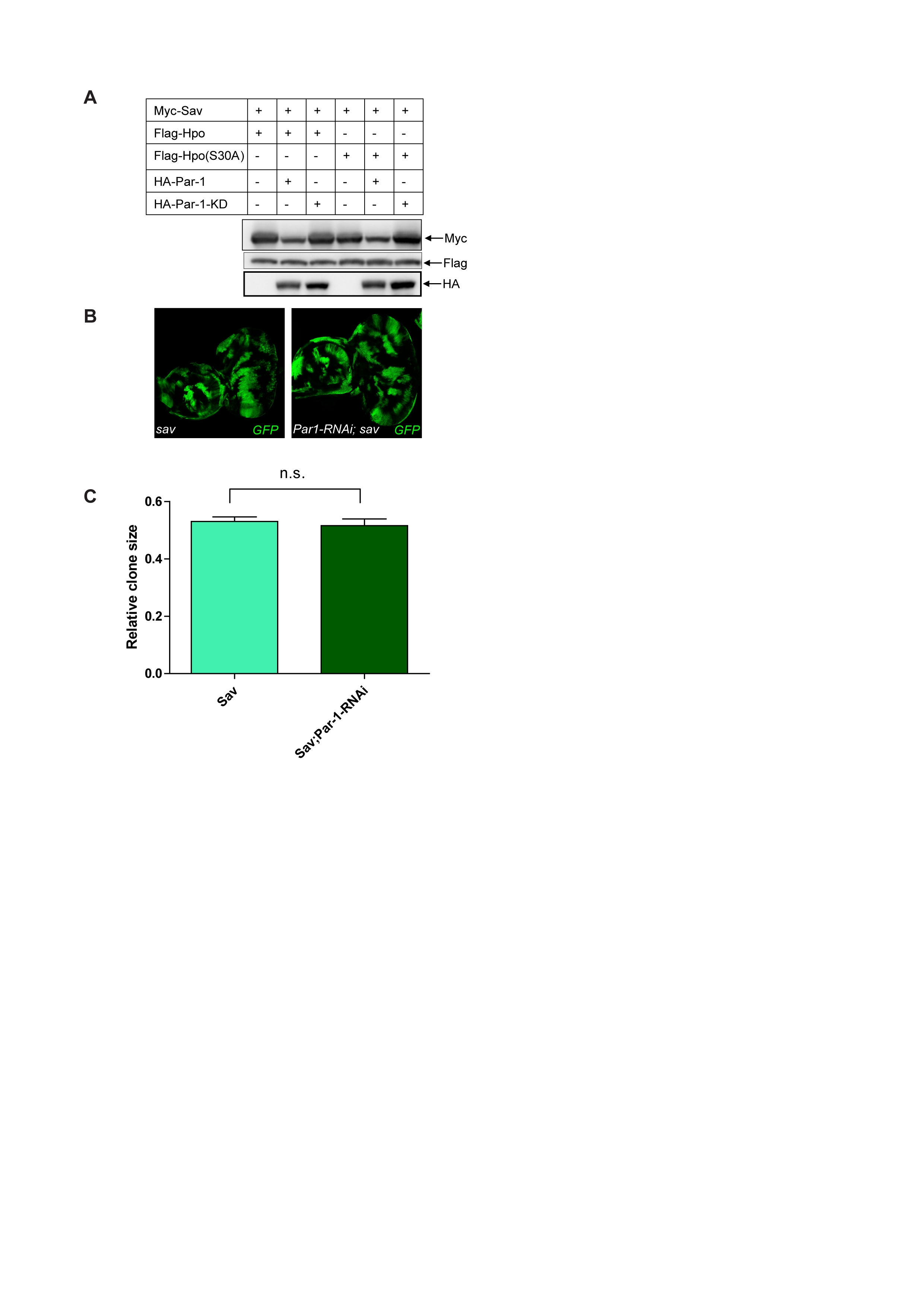

Supplement: Figure S6 — (A) Par-1 destabilizes Hpo-induced Sav accumulation independent of Par-1 phosphorylation at the Hpo Ser30 site. S2 cells were transfected with the indicated constructs followed by a Western blot analysis. Note that both wild-type Hpo and Hpo(S30A) could stabilize Sav. In addition, stabilized Sav can be destabilized by Par-1 overexpression. (B) par-1 functions upstream of sav in the Hpo pathway. Clones were generated using the MARCM system. The following genotypes were used: ey-flp, Ubi-Gal4, UAS-GFP; FRT82B SavSH13/FRT82B Gal80 (left panel), and eyflp, ubiGal4, UAS-GFP; Par-1-RNAi; FRT82B SavSH13/FRT82B Gal80 (right panel). (C) Quantification of the relative clone size. The relative clone size was calculated as the GFP area divided by the entire disc area. All of these data were expressed as the mean ± SEM. **p<0.01. **p<0.001. n>5, for each group. (TIF) [file pbio.1001620.s006.tif]

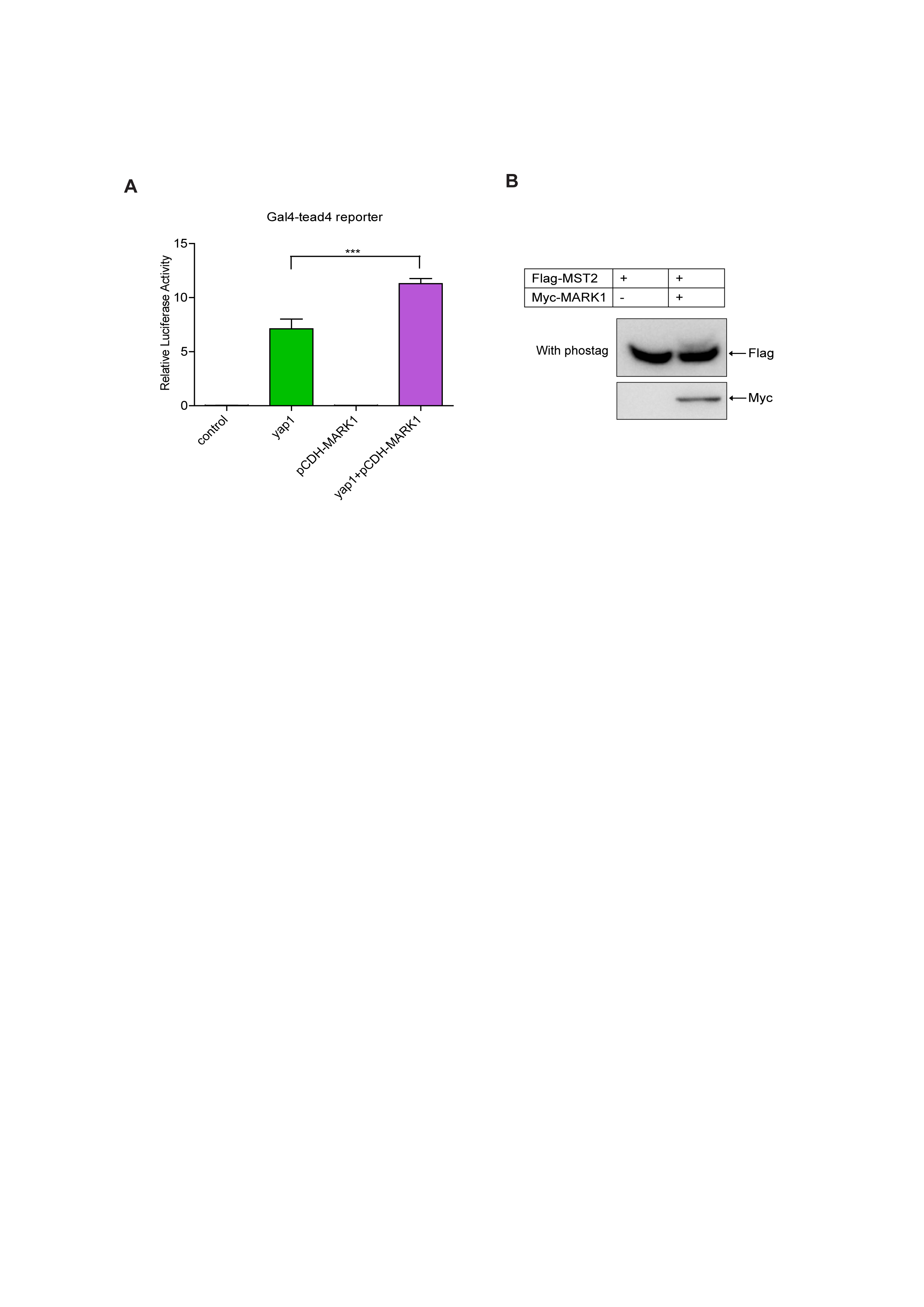

Supplement: Figure S7 — (A) MARK1 significantly enhances the transcriptional activity of Yap. HEK293T cells were transfected with the indicated plasmids, and the cell lysates were directly subjected to a dual luciferase reporter assay. Note that MARK1 synergized with Yap to promote the transcriptional activity of TEAD. (B) MARK1 induces a MST2 phosphorylation mobility shift. HEK293T cells were transfected with the indicated plasmids and cell lysates were directly subjected to a phosphorylation mobility shift assay. Note that MST2 showed a shift band by MARK1 coexpression. (TIF) [file pbio.1001620.s007.tif]

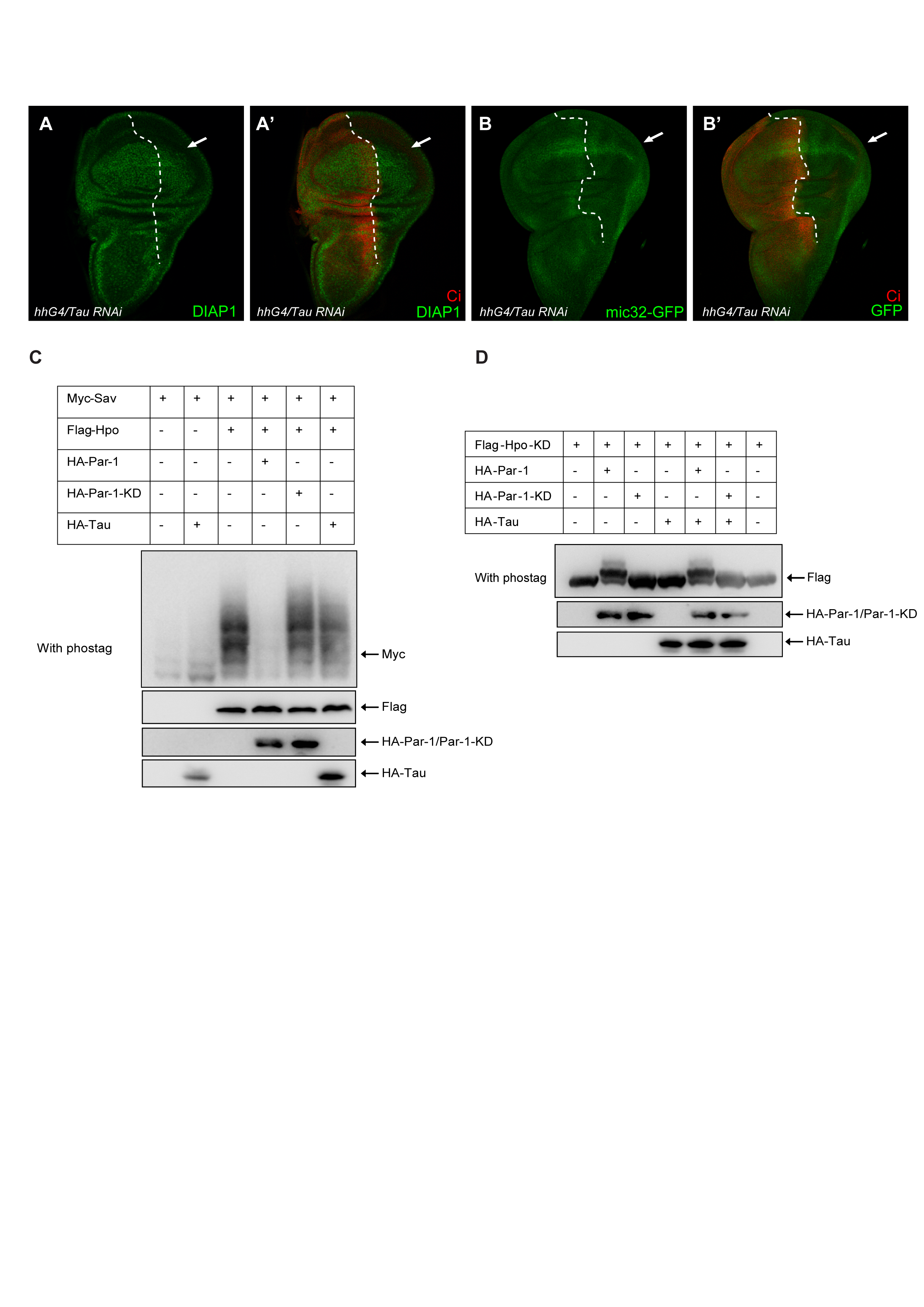

Supplement: Figure S8 — (A–B′) Wing discs expressing Tau RNAi (A–B′) with hh-Gal4 were immunostained with anti-Ci (red) and anti-DIAP1(green A–A′) to demonstrate the expression level of DIAP1 (A–A′) and mic32-GFP (B–B′). Note that the expression of Tau RNAi failed to affect Hpo pathway-responsive gene expression. The arrows indicate the P-compartment. (C–D) S2 cells were transfected with the indicated constructs followed by Western blot analyses. Note that Tau affects neither the phosphorylation status of Sav (C) nor the mobility shift of Hpo (D). (TIF) [file pbio.1001620.s008.tif]
